# Microbial control of mucosal wound immunity in colorectal anastomotic healing: from barrier repair to matrix failure

**DOI:** 10.3389/fimmu.2026.1928237

**Published:** 2026-10-21

**Authors:** Zhixuan Xu, Weipeng Chen, Feng Lu

**Affiliations:** Department of General Surgery, Binhai County People’s Hospital, Yancheng, Jiangsu, China

**Keywords:** epithelial restitution, gut microbiome, innate sensing, interleukin-22, matrix remodeling, mucosal immunity, neutrophils, wound healing

## Abstract

Colorectal anastomotic leakage is commonly framed as a technical complication or a consequence of intestinal dysbiosis. Neither view fully explains how a contaminated mucosal wound restores epithelial continuity while preserving a load-bearing submucosal matrix. We define anastomotic healing as a spatially organized mucosal immune process: luminal microbes and metabolites, the epithelial wound edge, recruited immune cells, fibroblasts, collagen, and vascular support occupy distinct but interacting compartments. The most compelling available anastomosis-specific evidence supports a destructive pathway in which expressed microbial virulence, host plasminogen activation, epithelial inflammatory signaling, sustained neutrophil recruitment, and MMP9 converge on collagen loss. A complementary repair pathway is supported by human-to-mouse microbiota-transfer and metabolite-rescue experiments, but this evidence is less direct and more heterogeneous. It links commensal function and AhR-dependent IL-22 signaling to epithelial restitution, myeloid resolution, and matrix repair. NLRP3, NLRP6, and NLRC4 remain candidate threshold regulators rather than demonstrated mediators of colorectal anastomotic healing. Human macrophage atlases refine cell-state definitions, but prospective evidence linking anastomotic macrophage states or efferocytosis to leakage remains limited. We propose an evidence-weighted framework that separates human association, direct microbial-function measurements, experimental causation, and human intervention evidence. Translation will require paired spatial sampling and functional microbial assays, together with immune, matrix, mechanical, target-engagement, and ecological-safety endpoints. This framework extends beyond taxonomic dysbiosis toward testable wound-immune endotypes and mechanism-matched prevention.

## 1 Introduction: colorectal anastomosis as a mucosal wound-immunity problem

Anastomotic leakage is among the most consequential complications of colorectal surgery. A localized failure of tissue repair can progress to pelvic sepsis, reoperation, prolonged hospitalization, permanent stoma, impaired quality of life, or death. Leakage is defined by pathological communication between the intra- and extraluminal compartments, but this clinical endpoint can arise through heterogeneous biological routes (1). Contemporary estimates remain approximately 3-6% after ileocolic anastomosis and 10-20% after low colorectal anastomosis, with variation related to anatomy, diversion, surveillance, and definition (2). Technical construction and perfusion establish the initial conditions, but they do not fully explain whether the wound will contain microbes, restore epithelial continuity, and rebuild load-bearing tissue.

A colorectal anastomosis is a full-thickness wound created at a densely colonized mucosal surface. Resection disrupts epithelial and mucus barriers, exposes submucosal collagen, releases damage-associated signals, and changes local oxygen and nutrient gradients. These changes allow luminal organisms and microbial products to reach immune and stromal compartments that are normally segregated from the intestinal lumen.

Successful healing therefore requires coordinated epithelial restitution, innate microbial containment, resolution of neutrophilic inflammation, macrophage-supported stromal repair, vascular and metabolic competence, and balanced matrix turnover. The same inflammatory and proteolytic systems that support debridement and host defense can become destructive when activation is excessive, spatially misdirected, or prolonged (3–5).

This perspective changes the role assigned to the microbiome. The relevant exposure is not a stable stool community labeled as “dysbiotic,” but a wound-expressed function at a newly created mucosal interface. Collagenolytic *Enterococcus faecalis* can activate host MMP9 and promote matrix failure, whereas *Fusobacterium nucleatum* can induce epithelial MMP9 and drive a NOD1-RIPK2-ERK–IL-1β–neutrophil proteolytic program (6–8). Conversely, uncomplicated-healing communities, prebiotic-supported microbial metabolism, and tryptophan-derived indoles can improve barrier or matrix repair in experimental systems (9–12). Microbial influence is therefore bidirectional: organisms can amplify tissue injury, but microbial metabolites and community functions can also increase mucosal repair reserve.

Human evidence remains difficult to reconcile because different specimens interrogate different biological compartments and phases of healing. Preoperative stool may capture susceptibility; intraoperative mucosa or stapled doughnuts are closer to the future wound; postoperative stool, drain fluid, or infection cultures may reflect an already evolving defect. Antibiotics, bowel preparation, diversion, tumor location, radiotherapy, diet, opioids, and institutional ecology further modify both microbial exposure and host repair. A 2026 systematic review identified only 11 comparative human studies comprising 551 participants, and only three reported functional analyses (13). The recurring taxonomic inconsistency should not be interpreted as evidence against microbial involvement. Rather, it shows that organism abundance alone is an inadequate proxy for strain-level virulence, metabolite production, tissue localization, or immune consequence.

Recent reviews have summarized clinical associations, perioperative microbiome modulation, and proposed molecular mechanisms (13–16). This review takes a different approach by treating the anastomotic interface as a spatial mucosal immune niche rather than a generic postoperative microbial ecosystem. It organizes evidence around temporally ordered wound-immune programs—containment, restitution, resolution, stromal repair, and matrix remodeling—while separating human association, anastomosis-specific causality, and indirect intestinal evidence. It also defines candidate immune pathways by the perturbation, rescue, and target-engagement evidence required to establish mediation, a distinction that is particularly important for inflammasomes.

Unlike previous reviews organized primarily around microbial taxa, perioperative dysbiosis, or candidate interventions, the present framework jointly considers tissue compartment, postoperative phase, and healing endpoint. This distinction helps explain why stool associations may not reflect wound-localized microbial activity, why restoration of epithelial continuity does not necessarily indicate recovery of load-bearing strength, and why the effect of an intervention may depend on whether it is applied during microbial containment, proliferative repair, or matrix remodeling. The framework therefore converts apparently conflicting findings into testable spatial and temporal hypotheses.

Accordingly, this review reframes colorectal anastomotic leakage as a failure of spatially organized mucosal wound immunity. It integrates a protective AhR–IL-22/barrier-repair route with a destructive microbial virulence-neutrophil-MMP9 route, and it positions inflammasomes as candidate threshold regulators that require pathway-specific validation. The goal is to move beyond taxonomic dysbiosis toward testable wound-immune endotypes, spatially resolved human studies, and mechanism-matched leak prevention.

## 2 Review approach

This article is a critical mechanistic narrative review. Literature was searched up to 28 June 2026 using PubMed/MEDLINE, with additional records identified through Europe PMC, OpenAlex, Crossref, and backward and forward citation searching. Searches focused on colorectal or intestinal anastomotic healing, anastomotic leakage, gut microbiome, microbial virulence, epithelial restitution, barrier immunity, innate sensing, neutrophils, macrophages, matrix remodeling, MMP9, IL-22, and perioperative interventions.

Representative PubMed/MEDLINE searches combined an anastomosis concept (“colorectal anastomosis”, “intestinal anastomotic healing”, or “anastomotic leakage”) with microbial and immune concepts (microbiome, microbiota, microbial virulence, epithelial restitution, neutrophil, macrophage, MMP9, IL-22, AhR, innate sensing, or inflammasome). Pathway-focused searches combined individual mediators with anastomosis, intestinal wound healing, epithelial repair, collagen remodeling, bursting pressure, or leakage. The complete representative search strings and date limits are provided in **Supplementary Methods**.

Key studies were selected when they directly measured anastomotic or peri-anastomotic tissue, tested a wound-relevant microbial function, included pathway perturbation or rescue, reported structural or mechanical healing, or provided prospective human or randomized intervention evidence. Studies from colitis, infection, organoids, superficial mucosal wounds, or other organs were retained only when they addressed a mechanistic gap that could not be illustrated with an anastomosis-specific study; such evidence is explicitly labeled indirect. No language, publication-year, or species restriction was applied during the mechanistic searches.

Evidence was interpreted according to the inference supported by each design. The hierarchy used in this review gives greatest clinical weight to randomized human interventions, large prospective cohorts with prespecified outcomes, functional human tissue studies, and externally validated prediction studies. Anastomosis-specific experiments with pathway perturbation, rescue, and structural or mechanical endpoints provide the strongest mechanistic evidence. Human taxonomic associations and small exploratory cohorts are hypothesis-generating unless they include direct measures of microbial function. Evidence from colitis, infection, organoids, superficial mucosal wounds, narrative reviews, or single-group experiments is labeled indirect or supportive when extrapolated to a full-thickness colorectal anastomosis. These categories are claim-specific rather than a single global score.

Causal language was used only when perturbation of a microbial or host pathway changed a structural healing or leakage-related phenotype, preferably with rescue or target-engagement evidence. Taxonomic detection was not treated as expressed virulence, and IL-1β elevation alone was not treated as inflammasome activation. Because this is a narrative review, SANRA informed clarity, search description, and evidence referencing; PRISMA was not applied as if a systematic review had been conducted (17, 18). Human microbiome reports were assessed against STORMS domains, animal-study interpretation considered ARRIVE 2.0, and prediction claims were judged using PROBAST principles (19–21). No pooled estimate was attempted because populations, compartments, assays, and outcomes were heterogeneous.

STORMS domains considered in human microbiome reports included sampling site and timing, perioperative exposures and clinical metadata, specimen handling, sequencing and bioinformatic methods, batch effects, compositional-data analysis, and outcome definition. ARRIVE 2.0 informed attention to the animal model, experimental unit, group size, randomization, blinding, exclusions, intervention timing, and structural or mechanical endpoints. PROBAST principles informed appraisal of participant selection, predictors, outcome definition, event counts, missing data, overfitting, internal and external validation, calibration, and clinical utility. These frameworks were used as structured reporting and interpretation lenses rather than formal numerical scores.

## 3 The colorectal anastomotic interface: a spatial mucosal immune niche

### 3.1 Layered wound biology determines immune exposure

The anastomosis is not a uniform tissue compartment. The epithelial surface, lamina propria, submucosal matrix, muscularis, serosa, mesenteric border, and peri-anastomotic fluid are exposed to different microbial pressures, oxygen gradients, mechanical forces, and immune-cell distributions. Sutures or staples provide initial apposition, but the submucosal extracellular matrix supplies much of the load-bearing strength. Layer-specific histology and transcriptomics show that mucosa/submucosa, muscularis, and serosa follow distinct yet coupled healing trajectories; disruption in one layer can therefore alter both immune access and mechanical competence in another (3–5).

The intact colon separates microbes from tissue through two MUC2-dependent mucus layers. The dense inner layer is normally attached to the epithelium and largely excludes bacteria, whereas the looser outer layer provides a habitat for commensals. Goblet cells continuously supply MUC2, and sentinel goblet cells at crypt openings can couple microbial sensing to coordinated mucin secretion (22, 23). Surgical transection disrupts this architecture precisely where staples or sutures must hold the bowel wall.

Below the mucus, polarized epithelial cells maintain selective permeability through apical junctional complexes and direct antimicrobial peptides and secretory products toward the lumen. The basement membrane anchors the epithelium and separates it from lamina propria immune and stromal cells. Successful healing must therefore restore more than surface coverage: it must re-establish polarity, tight-junction continuity, mucus organization, antimicrobial compartmentalization, basement-membrane attachment, and the mechanically competent submucosa.

The microbial niche is also spatially organized. In a rat model, the largest postoperative ecological shifts occurred in anastomotic tissue rather than luminal contents, with enrichment of tissue-associated facultative organisms and predicted adhesion, invasion, and proteolysis functions (24). This finding supports paired luminal and tissue sampling, but it does not mean that every postoperative compositional shift is pathogenic.

This layered anatomy gives barrier failure immunological and mechanical significance. An epithelial defect does more than increase permeability: it determines which microbial products reach pattern-recognition receptors, allows organisms and secreted enzymes to approach exposed collagen, and sustains neutrophil recruitment through continued microbial and damage signaling. Conversely, a mechanically weakened submucosa can enlarge epithelial separation and convert local inflammation into contamination and sepsis. Barrier failure and matrix failure are therefore coupled processes, but they are not interchangeable endpoints. Future studies should measure epithelial continuity, bacterial proximity, protease activity, collagen organization, and mechanical strength as related but distinct readouts.

### 3.2 Compartment and time constrain causal inference

Sampling site and timing determine the biological question. Preoperative stool may capture susceptibility but incompletely represents organisms attached to the future wound. Intraoperative mucosa, resection margins, stapled doughnuts, and tissue from each anastomotic end are anatomically closer to the wound and precede deterioration. Postoperative stool reflects fasting, transit changes, bowel preparation, and antibiotics; drain fluid or lavage samples may detect an evolving defect; and tissue obtained after leakage is vulnerable to contamination and reverse causation. Drain-fluid detection of *E. faecalis* can have early diagnostic value, but it does not establish that the detected organism initiated the defect (25).

Larger longitudinal cohorts reinforce this compartment- and context-dependence. Surgical procedure, center, bowel preparation, and diversion can explain substantial perioperative compositional variation, whereas global diversity differences between leak and non-leak groups are inconsistent (26–28). Experimental work also shows that bowel preparation can shift community composition without changing leakage, indicating that ecological disturbance is not equivalent to failed healing (29). A useful human biomarker must therefore specify its intended purpose: preoperative risk stratification, detection of an active postoperative mechanism, or recognition of an already evolving defect.

Human mucosal immune profiling is beginning to address this gap, but it remains anatomically indirect. In the multicenter IMMUNOREACT 4 analysis, flow cytometry of histologically normal tumor-adjacent rectal mucosa in a prospective cohort and immunohistochemistry in a larger retrospective cohort identified epithelial HLA upregulation and cytotoxic or T-helper-1-skewed lymphocyte features associated with later leakage. Discrimination was modest, and the sampled tissue was tumor-adjacent rather than the anastomotic interface. These findings therefore define a candidate mucosal susceptibility field, not a demonstrated immune cause of leakage at the healing wound (30).

Human microbiome studies also require explicit control of antibiotic exposure, diet, bowel preparation, tumor location, neoadjuvant treatment, comorbidity, specimen handling, extraction method, sequencing platform, bioinformatic pipeline, and batch. Relative-abundance data are compositional and cannot be interpreted as absolute expansion without appropriate analysis or quantitative anchoring (31). Prediction studies should report the number of leakage events, candidate-to-event ratio, internal validation, external validation, calibration, decision-curve or other clinical-utility measures, and confidence intervals rather than relying on the area under the receiver-operating characteristic curve alone.

STORMS was used as a reporting lens for human microbiome studies, not as a design-quality score (19). Leakage outcomes should be stratified by anastomotic level, clinical versus radiological detection, standardized severity grade, and duration of follow-up. These details are necessary to distinguish a biomarker of a clinically important leak from a signal driven by surveillance intensity or late contamination.

### 3.3 Surgical factors set the immune-injury threshold

Mucosal immune states are conditional on the wound environment. Ischemia and low oxygen limit epithelial metabolism and collagen maturation; altered pH, phosphate, iron, and exposed matrix proteins can change microbial behavior; and antibiotics, opioids, and NSAIDs reshape ecological and host responses. In experimental ischemia, MMP and TIMP activity extends beyond the immediate suture line, while pharmacological or genetic modulation of HIF-prolyl hydroxylases changes macrophage activity, collagen deposition, bursting pressure, and leakage (32, 33). Perfusion, microbial behavior, and host proteolysis should therefore be studied as interacting determinants rather than independent variables.

An immune-centered model must retain conventional clinical factors. Tissue tension, staple compression, technical defects, radiotherapy, bowel obstruction, anemia, hyperglycemia, malnutrition, corticosteroids, NSAIDs, contamination, vasopressor exposure, and prolonged surgery can alter the wound environment or reduce its capacity to tolerate microbial injury. In the prospective LekCheck cohort, low hemoglobin, contamination, hyperglycemia, operations longer than 3 hours, vasopressor use, suboptimal antibiotic timing, and epidural analgesia were associated with leakage (34). Mechanistic cohorts should report these variables and test effect modification rather than treat them only as nuisance covariates.

Perfusion correction alone is not uniformly sufficient. In AVOID, fluorescence-guided perfusion assessment did not significantly reduce 90-day clinically relevant leakage across colorectal operations; IntAct likewise did not meet its primary endpoint in rectal cancer, although its estimate favored fluorescence guidance (35, 36). These trials do not make perfusion unimportant. They show that leakage can persist despite one-time intraoperative assessment because tissue tension, evolving microvascular injury, inflammation, microbial function, and matrix failure remain active after reconstruction.

### 3.4 Evidence-weighted spatial model of anastomotic wound immunity

**Figure 1** introduces the colorectal anastomosis as a layered wound niche in which luminal microbes, the epithelial edge, lamina propria immune cells, the submucosal collagen matrix, and vascular support occupy coupled but non-equivalent compartments. Barrier integrity and load-bearing recovery are therefore evaluated as related but distinct outcomes. The principal compartments, repair tasks, failure-associated events, and informative measurements are summarized in **Table 1**.

**Figure 1:**
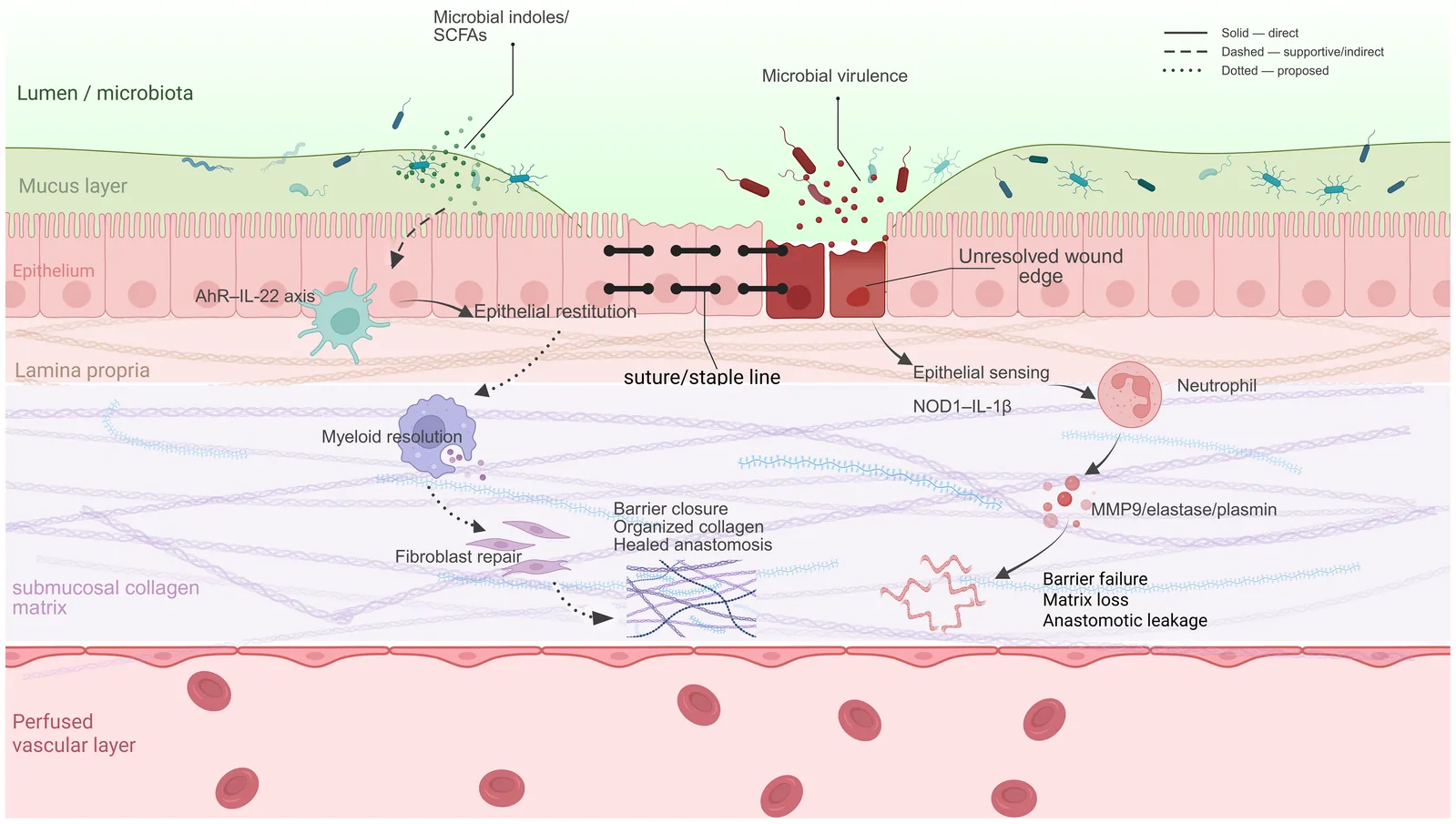
Spatial model of mucosal immune regulation in colorectal anastomotic healing. Luminal microbiota, the epithelial wound edge, lamina propria immune cells, the load-bearing submucosal collagen matrix, and vascular support are shown as coupled but distinct compartments. The suture/staple line is retained as a conceptual marker of bowel-wall approximation from the first-revision schematic rather than an anatomically scaled depiction of a specific hand-sewn or stapled technique. Its illustrated position does not imply mucosa-only engagement or define the actual depth, trajectory, or geometry of tissue capture, which varies with technique; tissue layers and device geometry are not drawn to scale. Arrows depict conceptual relationships and do not by themselves assign uniform evidentiary weight. Direct anastomosis-specific mechanisms, supportive or indirect evidence, and proposed links requiring validation are distinguished in Section 3.4 and **Table 2**. Epithelial repair, collagen organization, mechanical recovery, and clinical leakage are not interchangeable endpoints.

**Table 1:** Immune and repair compartments at the colorectal anastomotic interface.

Compartment or program	Principal repair task	Failure-associated immune or microbial event	Informative measurements	Current evidence
Epithelium and mucus	Rapid restitution, mucus recovery, microbial segregation	Persistent defect, microbial contact, epithelial inflammatory signaling, MMP9 induction	Spatial histology, epithelial continuity, MUC2, tight-junction proteins, bacterial proximity	Direct temporal anatomy; pathway-specific human data limited (3, 5)
Lamina propria innate compartment	Containment of translocated organisms and removal of debris	Sustained chemokine and cytokine signaling, excessive neutrophil influx	Multiplex imaging, flow or single-cell profiling, IL-1β, IL-22, neutrophil density	Direct animal pathways; sparse prospective human localization (8, 11)
Submucosal matrix	Preservation and replacement of load-bearing collagen	GelE/SprE activity, MMP9, elastase, plasminogen activation, collagen disorganization	Collagen I/III architecture, *in-situ* zymography, MMP/TIMP balance, breaking pressure	Most compelling available anastomosis-specific causal evidence (6, 7, 37)
Myeloid resolution program	Transition from antimicrobial inflammation to repair support	Prolonged neutrophilia or mistimed loss of reparative myeloid functions	Lineage-resolved spatial profiling, phagocytosis, cytokines, growth factors, temporal depletion	Direct timing evidence in mice; microbial mediation unresolved (38)
Stromal and vascular niche	Fibroblast matrix synthesis, organization, perfusion, angiogenesis	Reduced collagen synthesis, hypoxia, impaired vascular support	Fibroblast states, COL1A1/COL3A1, hydroxyproline, VEGFA, perfusion, mechanical testing	Direct repair anatomy; immune-microbial linkage incomplete (4, 39)
Luminal and tissue-associated microbiota	Colonization resistance and metabolite supply without tissue invasion	Virulence induction, collagenolysis, loss of repair-supportive metabolites	Paired stool and tissue metagenomics, metatranscriptomics, culture, enzyme assays, metabolomics	Human association plus direct experimental causal pathways; no universal taxon (13)

The table separates the major biological task of each compartment from candidate measurements and the strength of current evidence.

**Table 2:** Evidence map of immune pathways in colorectal anastomotic healing.

Immune or repair pathway	Anastomosis-specific evidence	Human evidence	Main limitation	Current interpretation
Microbial collagenase-host MMP9	Gene/function perturbation, bacterial elimination, host MMP9 suppression, structural rescue	Association of *E. faecalis*, collagenolytic capacity, or MMP9 with complications	Human function often inferred from taxonomy; late sampling	Most directly supported destructive microbial-host pathway (6, 54)
Epithelial MMP9 induction by *F. nucleatum*	Co-culture and anastomosis models with pathway interrogation and collagen loss	Limited tissue association	*In-vivo* rescue incomplete; modest samples	Supported epithelial route to matrix injury (7)
NOD1-RIPK2-ERK-IL-1β-neutrophil axis	Receptor knockdown, pathway inhibition, neutrophil perturbation, protease and collagen endpoints	No prospective spatial validation	Letter-format report; small groups; source of IL-1β not fully resolved	Supported candidate innate-neutrophil route (8)
AhR–IL-22 epithelial repair	Metabolite anchoring, genetic and pharmacological perturbation, rescue, leak endpoint	Human metabolite anchoring; no prevention trial	Context, dose, cellular source, and ecological effects require validation	Most directly resolved preclinical repair-supportive immune pathway (12)
Phase-specific myeloid/macrophage program	Timed CD11b-positive-cell depletion with epithelial, collagen, vascular, and mechanical endpoints	Prospective CD68/neutrophil tissue association and human reference atlases; no pathway-resolved healing cohort	Marker abundance is not cell state or mediation; human and murine states do not map one-to-one	Human susceptibility signal plus direct mouse timing and indirect TREM2/IL-10 repair hypotheses (38, 76–79)
NOD2-dependent local repair	Genetic deficiency impairs healing in mice	No direct human anastomotic validation	Direction and cell type require resolution	Supports protective, context-dependent innate sensing (67)
NLRP3, NLRP6, and NLRC4 inflammasomes	No qualifying pathway-specific anastomosis perturbation; indirect models suggest protective barrier effects and possible injury when activation is excessive	No prospective local assembly, cleavage, or cell-source data	DSS injury, biopsy wounds, and infection do not establish collagen-bearing wound repair; NLRP6 ecology is facility sensitive	Candidate, bidirectional innate-sensing tier; neither inhibition nor activation is clinically justified (68, 69, 71–73, 75)

Evidence levels reflect the specific pathway claim, not overall study quality.

The repair-supportive arm should not be interpreted as a single experimentally demonstrated causal cascade. Direct anastomosis-specific evidence supports parts of the metabolite-AhR-IL-22-epithelial repair pathway, whereas proposed connections to myeloid resolution, fibroblast activity, collagen organization, and mechanical recovery are assembled from separate studies and, in several instances, non-anastomotic injury models. The proteolytic arm contains more direct anastomosis-specific perturbation and rescue evidence, but even here the contribution of each pathway is conditional on organism, wound environment, and postoperative phase.

Accordingly, **Figure 1** distinguishes direct anastomosis-specific mechanisms, supportive or indirect evidence, and proposed links requiring validation. Improved epithelial repair is not treated as evidence of restored collagen organization or mechanical strength unless those endpoints were measured directly. Anastomosis-specific and supporting evidence for the major immune pathways is summarized in **Table 2**.

## 4 Epithelial restitution and barrier immunity

This section expands the epithelial and barrier compartment of the spatial model shown in **Figure 1**, focusing on how restitution limits immune exposure and how persistent barrier defects convert containment into proteolytic injury.

### 4.1 Restitution is an early immune-control mechanism

Epithelial repair has at least two temporally distinct components. First, surviving epithelial cells migrate across the denuded surface and form a provisional seal before crypt regeneration is complete. Later, crypt progenitor proliferation and differentiation replenish specialized epithelial lineages and restore mature barrier function. Collapsing these phases into a single proliferation or permeability measure obscures whether an intervention accelerates wound coverage, expands progenitors, or restores differentiated tissue.

Inflammatory and matrix-derived signals can create a temporary regenerative epithelial state. IL-22 activates epithelial STAT3 and supports mucosal wound healing; prostaglandin E2 drives wound-associated epithelial-cell differentiation; and extracellular-matrix remodeling can activate FAK/Src-YAP/TAZ signaling and fetal-like epithelial reprogramming (40–42). These studies largely use colitis, organoid, or mucosal-injury models, so their direct contribution to a mechanically loaded anastomosis remains to be tested.

Restitution should not be collapsed into a single “barrier integrity” score. Epithelial coverage, tight-junction abundance, mucus thickness, transepithelial permeability, bacterial proximity, and microbial translocation are related but non-equivalent readouts. A wound may regain partial epithelial coverage while junctions remain permeable, or preserve junctional proteins while mechanical separation exposes the submucosa. Future studies should therefore combine structural microscopy, permeability assays, mucus and tight-junction markers, and bacterial-localization measurements to distinguish epithelial closure from functional microbial containment.

### 4.2 Barrier failure can convert containment into proteolytic inflammation

Persistent epithelial defects increase exposure of lamina propria and submucosal cells to PAMPs, DAMPs, bacterial enzymes, and host substrates. This sustains chemokine production and neutrophil recruitment while allowing secreted proteases to approach the load-bearing submucosal matrix. *F. nucleatum* provides a pathway-specific example: epithelial E-cadherin/β-catenin signaling and NOD1-RIPK2-ERK activation link microbial contact to IL-1β-associated neutrophil recruitment and collagen injury in experimental anastomoses (7, 8). The matrix effectors are discussed in Section 5; the key point here is that persistent barrier failure changes both immune access and enzymatic access to the submucosa.

This sequence is better understood as a feed-forward loop than as a one-way barrier-to-leak cascade. Initial epithelial injury increases microbial contact; epithelial and myeloid signaling amplify proteolysis; matrix loss widens the defect; and local contamination further intensifies inflammation. Once clinical leakage is established, antibiotics, sepsis, fasting, drainage, and altered transit can profoundly reshape the microbiome, making late samples poor evidence of initiation. Sampling must therefore precede overt failure if epithelial immune signaling is to be interpreted as a causal contributor rather than a consequence of leakage (13, 26).

### 4.3 Commensal signals actively support barrier repair

Protective barrier immunity is an active repair program. Commensal recognition through Toll-like receptors and MyD88 can maintain epithelial homeostasis and protect against injury, while a wound-associated mucosal community can be selected by the local injury environment and stimulate epithelial migration (43, 44). These observations caution against treating microbial sensing as uniformly inflammatory.

Anastomosis-specific and transmural-injury studies support the same bidirectionality. Conventionally colonized rats developed stronger intestinal anastomoses than germ-free animals in one experiment, although effects depended on the colonizing organism (45). In a colonic transmural-defect model, *A. muciniphila* improved histological healing and reduced peritonitis and mortality through an intestinal epithelial MyD88-dependent mechanism associated with IL-22; the study also included short-term human mucosal exposure but did not test clinical leak prevention (46).

Human-to-mouse microbiota-transfer studies provide additional evidence that communities from uncomplicated healing can improve experimental barrier and matrix outcomes, whereas leak-associated donor communities can transmit impaired healing (11, 39). These experiments demonstrate transferable function but do not identify a universal protective consortium.

The AhR–IL-22 pathway is the clearest mechanistically resolved repair-supportive route. Tryptophan-derived indole metabolites reduced experimental leakage through AhR-dependent IL-22 production, with genetic, pharmacological, and rescue evidence supporting pathway involvement (12). Indirect organoid and injury studies define the boundary of this interpretation: IL-22 can support intestinal stem-cell-mediated regeneration, but prolonged or differently timed signaling may expand transit-amplifying cells while depleting Lgr5-positive stem cells (47, 48). AhR–IL-22 signaling should therefore be treated as a source-, dose-, and phase-dependent repair pathway. Translation requires local metabolite exposure, AhR target engagement, IL-22-producing cells, epithelial response, structural healing, and tumor-related safety rather than a circulating IL-22 measurement alone.

A recent mouse gastrointestinal-anastomosis study supports the same repair axis in a local-delivery context. A small-intestinal-submucosa bio-patch increased IL-22-positive ILC3s, ZO-1 and occludin, altered bacterial and fungal communities, and improved histological healing (49). Because the patch simultaneously affected immune cells, barrier proteins, microbiota, and metabolites, it does not isolate an ILC3/IL-22 mechanism as cleanly as receptor-level perturbation. It nevertheless supports local biomaterials as platforms for coordinated immune and ecological repair.

When barrier failure persists, microbial products and secreted enzymes gain access to the submucosal matrix, linking epithelial injury to the proteolytic mechanisms discussed next.

## 5 Neutrophil-MMP9 proteolysis and inflammatory matrix injury

This section dissects the proteolytic injury arm of **Figure 1**, in which expressed microbial virulence, epithelial inflammatory sensing, neutrophil recruitment, and host proteases converge on submucosal collagen loss.

### 5.1 Physiological matrix turnover creates a vulnerable interval

Matrix repair begins with a provisional fibrin-fibronectin scaffold that supports inflammatory-cell and fibroblast recruitment. Fibroblasts and myofibroblasts then deposit collagen under signals that include TGF-β, after which fiber organization, enzymatic cross-linking, and maturation restore load-bearing strength. The submucosa is especially important because it holds sutures or staples and supplies much of the bowel wall’s mechanical competence. Human data link lower collagen I/III ratios and higher mucosal or submucosal MMP expression with subsequent leakage, while microdissection shows that collagen loss and MMP activity concentrate in the suture-holding zone (50, 51). A human fibroblast model further shows site-dependent collagen deposition and responsiveness to TGF-β1 (52).

Formation and degradation overlap. Early collagenolysis removes injured matrix, but newly synthesized collagen is initially sparse and weakly organized. Selective inhibition of MMP-8, MMP-9, and MMP-12 improved breaking strength and reduced leakage in an obstructed-colon model, supporting a causal contribution of excessive proteolysis in that context (53). Because MMP activity is also required for cell migration and normal remodeling, this experiment supports selective, phase-specific investigation rather than routine clinical MMP inhibition.

MMP9 is important because it lies at the intersection of epithelial injury, neutrophil activation, microbial virulence, and matrix turnover. It can be produced or activated in several compartments and contributes to cell migration and normal remodeling as well as tissue injury. Human studies summarized in a systematic review generally associated higher MMP9 with leakage or postoperative complications, but specimen type, assay, timing, and endpoint varied, and not all findings were concordant (54). MMP9 should therefore be interpreted as a context-dependent effector within a local proteolytic network, not as a stand-alone diagnostic marker or a constitutively harmful molecule.

### 5.2 Microbial virulence can recruit host proteases

The most compelling available microbiome-to-leak mechanism begins with expressed collagenolytic virulence at the wound. In a defined model, E. faecalis GelE/SprE activity recruited host MMP9, degraded collagen, and promoted leakage; bacterial elimination or MMP9 suppression attenuated failure (6). Human cultures and sequencing have identified E. faecalis or putatively collagenolytic organisms in some leak samples, but most studies infer function from taxonomy rather than measuring enzyme activity (55–57). This distinction is critical. Commensal carriage does not predict expressed virulence: isolates differ in collagenolytic capacity, surgical injury can induce local wound programs, and wound-conditioned P. aeruginosa can become leak-promoting in sensitized animals (58, 59). The reproducible exposure is therefore an expressed wound function, such as collagenase activity, rather than a universal bacterial taxon. A prospective human study that combined stool and mucosal sampling with culture, virulence-gene analysis, and direct collagenase/protease assays associated functional Enterococcus isolates at surgical margins with leakage; fecal profiles were less informative (60). This design is more mechanistically informative than taxonomic abundance alone, but its small number of leakage events and lack of external validation limit predictive claims.

Host fibrinolysis provides a second amplification route. E. faecalis can exploit plasminogen to increase collagenolysis, and local tranexamic acid reduced pathogen-mediated leakage in mice (37, 61). Anti-virulence compounds that suppress collagenase production, including polyphosphate-based approaches, also prevent leakage in sensitized animal models without simply eradicating the organism (62, 63). These studies are important because they perturb a microbial or host function and recover a structural phenotype. However, model dependence, uncertain human target prevalence, and absent local target-engagement data preclude routine clinical use.

### 5.3 Neutrophils are necessary defenders and potential matrix effectors

Neutrophils provide rapid antimicrobial containment, phagocytosis, and debris clearance, but persistent recruitment can expose the wound to elastase, reactive oxygen species, MMPs, and extracellular traps. The pathological state is not neutrophil recruitment itself, but failure to terminate protease-rich neutrophilic inflammation after microbial containment has been achieved. The NOD1-RIPK2-ERK-IL-1β study provides an anastomosis-specific route from epithelial sensing to neutrophil chemotaxis, protease activity, collagen degradation, and leakage, with pathway and cellular perturbation reducing components of the phenotype (8). These data support a spatially sustained proteolytic-neutrophil state, not the claim that neutrophils are uniformly harmful or that systemic depletion would be safe. A translational endotype should therefore pair local neutrophil activation and MMP9 or elastase activity with collagen disorganization, while also demonstrating preserved antimicrobial defense after selective intervention.

Indirect mucosal-wound studies reinforce this duality. Soluble JAML shed from transmigrating neutrophils inhibited epithelial proliferation and wound repair, whereas post-migrated neutrophil engagement of epithelial ICAM-1 activated Akt/β-catenin signaling and accelerated biopsy-wound closure (64, 65). Neutrophil-specific CD45 deletion further impaired CD11b/Lyn signaling, antimicrobial activity, recovery from DSS injury, and biopsy-wound repair (66). These studies do not prove that the same pathways operate in an anastomosis, but they show why neutrophil abundance alone cannot distinguish protective containment from tissue-damaging persistence. The relevant measurement is therefore not neutrophil number alone, but the timing, localization, activation state, protease output, and resolution of the neutrophilic response.

### 5.4 The therapeutic target is excessive local proteolysis, not maximal inhibition

Microbial proteases, MMP9, neutrophil elastase, and plasmin can converge on matrix loss, but proteolysis is also required for debridement, epithelial migration, immune-cell trafficking, and collagen remodeling. The therapeutic goal is therefore not maximal inhibition. Broad or mistimed blockade could preserve damaged matrix, impair restitution, or compromise antimicrobial defense. The more relevant question is whether a defined proteolytic function is excessive in a specific compartment and postoperative window.

Useful pharmacodynamic endpoints include microbial collagenase activity, *in-situ* gelatinase activity, MMP/TIMP balance, neutrophil elastase, collagen I/III organization, hydroxyproline, bacterial burden, epithelial continuity, and mechanical strength. Leak rate alone cannot show that a mechanism was engaged. Conversely, reduced MMP9 without restored tissue architecture is not sufficient evidence of successful repair. A credible anti-proteolytic strategy should therefore demonstrate target engagement, preserved antimicrobial containment, restored matrix organization, and improved mechanical competence.

## 6 Innate sensing at the anastomotic wound: evidence, candidates, and limits

Innate sensing provides the decision layer between microbial containment and inflammatory amplification in **Figure 1**. The key issue is not whether pattern-recognition pathways are activated after surgery, but whether a specific sensor changes epithelial repair, neutrophil proteolysis, matrix integrity, or leakage.

### 6.1 Pathway-specific evidence must separate sensing from generic inflammation

Anastomotic surgery creates abundant inputs for innate sensing, including bacterial peptidoglycan and flagellin, extracellular ATP, ionic flux, hypoxia, reactive oxygen species, matrix fragments, and intracellular contents released from injured cells. These signals make NOD-like receptors, Toll-like receptors, and inflammasomes biologically plausible regulators of the healing interface. Plausibility, however, is not equivalent to mediation. A pathway-specific claim requires receptor or adaptor activation in the relevant cell type, appropriate downstream processing, temporal precedence, a structural phenotype after loss-of-function, and preferably rescue or target-engagement evidence.

This evidentiary standard is especially important for inflammasomes. Increased IL-1β can reflect transcriptional priming, altered secretion, reduced clearance, or non-canonical signaling rather than canonical inflammasome assembly. Evidence for NLRP3, NLRP6, or NLRC4 should therefore include pathway-specific readouts such as inflammasome assembly, caspase-1 processing, mature IL-1β or IL-18, gasdermin-D cleavage, pyroptosis, cell localization, and cell-specific perturbation. Without these measurements, “inflammasome activation” overstates the evidence and risks converting a plausible sensing hypothesis into an unsupported causal mechanism.

### 6.2 NOD1 has direct mechanistic support; NOD2 shows local repair relevance

Among candidate intracellular sensors, NOD1 currently has the clearest anastomosis-specific pathway evidence. In a mouse model and epithelial-neutrophil systems, F. nucleatum activated NOD1-RIPK2-ERK signaling, increased IL-1β-associated neutrophil recruitment and protease activity, and worsened collagen loss and leakage; pathway perturbation reduced components of the phenotype (8). This evidence is mechanistically informative, but it comes from a recent letter-format report with small experimental groups and no prospective human validation. NOD1 should therefore be treated as a supported candidate injury route, not as a settled clinical endotype.

NOD2 points in a different direction. NOD2 deficiency impairs ileocolonic anastomotic healing through local mechanisms in mice, supporting the broader concept that intracellular bacterial sensing can also promote repair (67). The contrast between NOD1-associated injury and NOD2-dependent repair cautions against viewing innate sensing as uniformly destructive. Future experiments should define the relevant cell type, ligand source, timing, and structural endpoint before assigning a sensor to either a pro-healing or pro-leak pathway.

### 6.3 Inflammasomes may regulate containment and inflammatory thresholds

NLRP3, NLRP6, and NLRC4 are best interpreted as candidate threshold regulators rather than direct collagen-degrading or matrix-repair effectors. Indirect models support plausible gatekeeper roles: NLRP3-ASC-caspase-1-IL-18 signaling can preserve epithelial integrity in DSS colitis; NLRP6 has been linked to biopsy-wound closure and mucus segregation, although microbiota-shaping effects appear facility-sensitive; and epithelial NAIP-NLRC4 can promote infected-cell expulsion and barrier-preserving contraction (68–75). At an anastomosis, these pathways could support early microbial containment, IL-18-dependent epithelial defense, and barrier maintenance. If activation is prolonged, excessive, or spatially misdirected, however, they could also amplify IL-1β release, pyroptosis, neutrophil recruitment, and protease exposure.

The main limitation is model fidelity. DSS injury, infection, and superficial biopsy wounds do not reproduce a full-thickness, collagen-bearing anastomosis exposed to mechanical load, ischemia, surgical tension, and luminal microbial pressure. Advancement requires epithelial- and myeloid-specific perturbation in anastomosis models, preferably under defined surgical modifiers, with time-resolved assessment of caspase-1 and gasdermin-D processing, mature IL-18/IL-1β localization, epithelial restitution, collagen organization, mechanical strength, and rescue. Until such data are available, inflammasomes should guide threshold-focused hypotheses rather than justify activation or inhibition as leak-prevention therapy.

## 7 Macrophage timing, stromal repair, and immune-resolution programs

This section expands the myeloid-resolution and stromal-support components of **Figure 1**. The central question is whether antimicrobial inflammation transitions into efferocytosis, macrophage-supported epithelial repair, fibroblast activation, vascular support, and organized collagen replacement at the appropriate time.

### 7.1 Repair depends on myeloid timing rather than a binary polarization label

Macrophages and related mononuclear phagocytes remove debris, coordinate antimicrobial responses, regulate neutrophil persistence, and communicate with epithelial, stromal, and vascular compartments. Human activation transcriptomes and intestinal atlases describe a spectrum of stimulus- and niche-dependent states rather than a stable M1/M2 or iNOS/CD206 dichotomy (78, 80). The clinically relevant question is therefore not whether macrophages are “pro-inflammatory” or “reparative” in general, but whether antimicrobial states transition toward resolution and tissue repair at the appropriate time while preserving local defense.

Evidence from skeletal muscle regeneration further illustrates that macrophage phenotypes and metabolic programs change across repair phases, although this cross-tissue evidence should not be treated as an anastomosis-specific mechanism (81).

Phase-specific depletion experiments provide direct evidence that timing matters. Depletion of CD11b-positive cells during distinct inflammatory, proliferative, or reparative windows altered re-epithelialization, collagen deposition, angiogenesis, transcriptomic programs, and bursting pressure in mouse anastomoses (38). Because CD11b depletion is not macrophage specific, these data do not define a single reparative macrophage lineage. They do show, however, that perturbing the same broad myeloid compartment at different phases can produce different structural consequences.

Colonic biopsy-wound studies provide lineage and mediator hypotheses that can now be tested in anastomotic models. TREM2 deficiency delayed wound closure and shifted the wound bed toward inflammatory cytokines and reduced epithelial proliferation, whereas conditional evidence implicated macrophage-derived IL-10 in epithelial CREB-WISP1 signaling, proliferation, and wound closure (76, 77). These mechanisms are spatially relevant to mucosal repair, but they remain indirect because superficial biopsy wounds do not reproduce a full-thickness, mechanically loaded anastomosis.

Direct negative evidence is equally important. In mice with Crohn’s-like ileitis, perioperative α4β7-integrin blockade changed immune and mesenchymal cell infiltration in ileocecal anastomoses but did not impair bursting pressure, matrix measures, or leak rate (82). This result shows that an immune-cell shift is not automatically a functional healing defect. Macrophage and myeloid studies should therefore pair immunophenotyping with epithelial closure, efferocytosis, collagen architecture, vascular support, and mechanical endpoints.

### 7.2 Human intestinal maps define candidate states, not anastomotic macrophage mechanisms

Direct human tissue evidence is limited but not absent. In a prospective cohort of 83 patients undergoing laparoscopic sigmoid resection for diverticulitis, seven developed leakage; matched-pair analysis found higher CD68-positive macrophage and neutrophil infiltration in mucosal and submucosal colonic wall obtained at the index operation (79). The low event count, marker-only phenotyping, and absence of serial wound sampling make this a susceptibility association rather than evidence of a macrophage mechanism. IMMUNOREACT 4 similarly linked epithelial HLA upregulation and lymphocyte features in tumor-adjacent rectal mucosa to later leakage, but did not resolve macrophage lineages or sample the healing interface (30). No identified prospective cohort has longitudinally measured anastomotic macrophage states, efferocytosis of apoptotic neutrophils, or TREM2/IL-10-related repair programs before leakage.

Human reference atlases nevertheless define the cell states that future anastomotic studies should interrogate. Duodenal transplant and normal-colon resection studies identify monocyte-like, inflammatory, antigen-presenting/phagocytic, subepithelial, submucosal, vascular-associated, and muscularis macrophage states with distinct tissue niches and replacement kinetics (78, 83). Candidate human analogues of reparative TREM2/IL-10-associated programs are most likely to reside among mature phagocytic, subepithelial, or submucosal macrophage states with high efferocytic, IL-10-related, and tissue-remodeling signatures. However, CD206-positive human colonic macrophages can be more mature and IL-10-high than CD206-negative cells, yet overlapping transcriptional programs prevent CD206 from serving as a stand-alone reparative label (84). Regional multiplexed imaging further shows that immune organization varies along the human intestine (85). Mouse CD11b depletion, F4/80/Arg1/iNOS labeling, and TREM2 or IL-10 perturbation therefore define testable hypotheses, not one-to-one human cell-state equivalence.

Disease atlases add another boundary. Spatial single-cell analysis in human inflammatory bowel disease resolves macrophage-neutrophil heterogeneity and macrophage-fibroblast communication, but chronically inflamed mucosa is not an acute, mechanically loaded postoperative wound (86). A human anastomosis study should therefore pair lineage-resolved spatial states with bacterial proximity, efferocytic cargo or receptor engagement, epithelial gaps, collagen organization, perfusion, and outcome over time. **Table 3** summarizes the translational mismatch that should constrain interpretation.

**Table 3:** Human-to-rodent translational boundary for macrophage evidence.

Domain	Rodent evidence commonly used	Human evidence currently available	Consequence for anastomosis claims
Cell identity	CD11b, F4/80, CX3CR1, Arg1/iNOS, or CD206-defined compartments	Multiple lamina propria, submucosal, vascular, and muscularis states defined by single-cell and *in-situ* mapping	Avoid assigning a human reparative lineage from one or two markers
Ontogeny and turnover	Variable embryonic and monocyte contributions across layers	Transplant and resection data support monocyte replacement with slower mature subsets, but lineage correspondence is incomplete	Treat depletion and lineage-tracing results as model-specific
Repair mediators	TREM2, macrophage IL-10/WISP1, COX2-dependent efferocytosis, and timed depletion alter murine repair	No prospective human anastomotic perturbation or pathway-resolved cohort	Describe these as testable pathways, not human leak mechanisms
Spatial context	Full-thickness experimental anastomoses, DSS injury, infection, or superficial biopsy wounds	Healthy resection margins, tumor-adjacent mucosa, and chronic IBD atlases	Require validation at the healing interface before leakage
Functional endpoint	Epithelial closure, collagen, angiogenesis, bursting pressure, or leak	Predominantly cell abundance, transcriptomic state, or association	Pair spatial state with efferocytosis, matrix architecture, and mechanical or clinical outcome

### 7.3 Neutrophil resolution and macrophage efferocytosis are linked repair decisions

Persistent neutrophilia can reflect ongoing microbial exposure, defective barrier closure, impaired clearance of dying cells, or sustained chemokine signaling. Macrophage efferocytosis and neutrophil activation should therefore be studied as linked repair decisions rather than separate cell-count variables. No human anastomosis study identified here has measured neutrophil persistence, apoptotic-cell uptake, and macrophage repair state in the same tissue over time, and direct microbiome-to-macrophage causal experiments remain limited.

Indirect mechanistic studies support resolution as an active repair program. Macrophage COX2 was required for efficient efferocytosis and for an efferocytosis-induced epithelial restitution phenotype in intestinal organoids, while locally generated resolvin E1 accelerated colonic biopsy-wound healing and epithelial migration (87, 88). These studies do not establish an anastomotic mechanism, but they provide measurable candidates—efferocytosis, lipid mediators, IL-10-related repair signaling, and epithelial restitution—for time-resolved surgical studies.

Community-transfer work offers a useful entry point because leak-associated and non-leak-associated donor microbiota produce different mucosal cytokine, barrier, and matrix phenotypes in antibiotic-conditioned recipients (11). The missing link is the mediating myeloid state. Spatial single-cell or multiplex imaging should therefore be coupled to efferocytic cargo, receptor engagement, bacterial proximity, epithelial gaps, neutrophil activation, fibroblast state, and collagen organization rather than used as descriptive clustering alone.

### 7.4 Stromal repair must be measured as structure and function

Fibroblasts rebuild the submucosal scaffold, but collagen quantity alone does not establish mechanical competence. A provisional fibrin-fibronectin matrix, fibroblast recruitment, myofibroblast differentiation, TGF-β signaling, collagen deposition, fiber organization, and cross-link maturation are separable processes. Hydroxyproline estimates collagen quantity; it does not measure fiber alignment, cross-linking, collagen type, or spatial continuity. Microbiota from patients without leakage can promote collagen synthesis in experimental systems, but the epithelial-stromal signals that connect microbial function to durable matrix restoration remain incompletely defined (11, 39, 52).

Perfusion provides essential context for stromal and immune repair. Oxygen supports epithelial metabolism, collagen maturation, and antimicrobial defense, while ischemia changes MMP/TIMP distribution and may alter pH, nutrient availability, microbial virulence, and immune-cell function (32). Experimental PHD inhibition improved healing in ischemic or septic anastomoses together with changes in macrophage state, collagen content, inflammation, and bursting pressure (33). These data support an interacting perfusion-immune-matrix model, not a claim that HIF manipulation is established therapy.

Recent biomaterial work illustrates how such paired endpoints can be integrated. In a rabbit gastrointestinal-anastomosis model, phase-adaptive VEGF-coated porous staples increased neovascularization, shifted macrophage markers toward a reparative phenotype, improved collagen organization, and increased bursting pressure (89). Because VEGF delivery, surface architecture, angiogenesis, and macrophage state changed together, the dominant mediator remains unresolved. The study is best interpreted as preclinical evidence for coordinated vascular-immune-matrix repair, not as proof of a single VEGF- or macrophage-dependent mechanism.

### 7.5 Healing endpoints are not interchangeable

Hydroxyproline content, histological collagen staining, bursting pressure, and breaking strength answer different questions. Hydroxyproline estimates collagen quantity but not organization or cross-link quality. Bursting pressure measures resistance of a closed intestinal segment to intraluminal pressure and is informative only while failure occurs at the anastomosis; breaking strength measures tensile load across an opened specimen and has a different time window and failure mode (90). Studies should report where failure occurred and should combine biochemical, architectural, and mechanical endpoints rather than treat them as interchangeable.

These stromal and vascular programs are influenced by microbial community function and metabolite availability, which are considered in the next section.

## 8 Repair-supportive microbial signals: AhR–IL-22, microbial metabolites, and mucosal repair reserve

Having defined the epithelial, myeloid, and stromal repair modules, this section returns to the upstream microbial functions that may increase mucosal repair reserve in **Figure 1**.

### 8.1 Microbial communities can increase or reduce repair reserve

The microbiome can shape the amount of injury that the anastomotic wound tolerates before structural failure. A repair-supportive community may maintain colonization resistance, supply metabolites used by epithelial and immune cells, limit expansion of collagenolytic organisms, and support balanced cytokine and stromal programs. In experimental models, low-fat/high-fiber dietary backgrounds and perioperative prebiotic supplementation improve healing, whereas high-fat/low-fiber exposure and selected drug interactions can worsen leakage-related phenotypes (9, 10, 91, 92).

These findings should not be interpreted as evidence that “more fiber” or any single community profile will prevent human leakage. Diet composition, obstruction risk, bowel preparation, perioperative fasting, antibiotics, and adherence determine whether a substrate reaches the relevant microbiota and whether the intended metabolite or microbial function is produced. Translation therefore requires a defined formulation, local exposure assessment, and confirmation of the intended functional response in the peri-anastomotic compartment.

### 8.2 AhR–IL-22 is a model for mechanism-matched metabolite research

As detailed in Section 4.3, the metabolite-AhR-IL-22 axis is the most pathway-resolved repair-supportive example identified in an anastomosis model. Its translational value lies in the causal design linking local exposure, receptor engagement, cytokine source, epithelial response, and structural outcome, not in assuming that every AhR ligand or sustained IL-22 signal is protective. Human translation therefore requires compound-specific exposure, target engagement, tumor-related safety, and compatibility with perioperative antimicrobial care.

### 8.3 SCFA-associated effects depend on epithelial location and condition

Short-chain fatty acids have compartment- and state-dependent effects. Butyrate oxidation by differentiated colonocytes increases oxygen consumption and stabilizes epithelial HIF, supporting barrier function (93). The same metabolite can inhibit proliferation when mucosal injury exposes crypt progenitors that are normally shielded by differentiated colonocytes (94). Therefore, local concentration, epithelial integrity, anatomical site, and timing determine whether butyrate is supportive, neutral, or inhibitory. Fiber intake or genus abundance should not be used as a surrogate for this functional exposure without metabolomics and pathway readouts.

FMT is useful for demonstrating that a transferable community phenotype exists, as shown by human-to-mouse studies using leak-associated and uncomplicated-healing donors (11). It is not yet a defensible perioperative therapy because donor variability, pathogen and resistance-gene transmission, uncertain engraftment, interaction with prophylactic antibiotics, and postoperative translocation risk remain unresolved. Defined metabolites, rational consortia, or functionally selected strains are more reproducible translational candidates, but each still requires confirmation of local target engagement, ecological safety, and compatibility with standard perioperative care.

### 8.4 Lymphoid and adaptive immune pathways are plausible but indirect

IL-22 activity depends on timing, concentration, cellular source, and epithelial target state. CX3CR1-positive mononuclear phagocytes can support ILC3 production of IL-22, whereas ILC3s can also promote YAP-dependent epithelial repair independently of IL-22 (95, 96). ILC2-derived amphiregulin can support intestinal tissue protection through EGFR signaling, while expansion of interferon-associated B-cell states can impede stromal-epithelial regenerative communication (97, 98). These pathways derive mainly from colitis or general mucosal-injury models and should be treated as candidate mechanisms for anastomotic study.

Regulatory T cells, Th17 cells, γδ T cells, secretory IgA, and B-cell effects on regenerative niches may further influence microbial containment and repair. Direct anastomosis-specific perturbation evidence for these pathways was not identified in this review. Their inclusion in future spatial studies should therefore be hypothesis-driven and paired with epithelial, microbial-function, matrix, and mechanical endpoints.

### 8.5 Mycobiome and virome evidence is uneven

The microbial compartment is not limited to bacteria. Commensal fungi signal through receptors such as Dectin-1, and Debaryomyces hansenii can colonize injured intestinal tissue and impair mucosal healing through a myeloid type I interferon-CCL5 pathway in Crohn’s disease tissue and mouse wound models (99, 100). These findings establish biological plausibility for fungal effects but do not demonstrate an anastomotic mycobiome mechanism.

Direct evidence for the anastomotic virome and bacteriophages remains sparse. Viral and phage studies should distinguish baseline ecological susceptibility from postoperative expansion, horizontal gene transfer, and effects secondary to antibiotics or leakage. Until such data are available, the broader term microbial is retained to identify this evidence gap rather than imply equivalent evidence across bacteria, fungi, and viruses.

Together, these microbial and metabolite data support a repair-supportive arm of the model, but they also show why local exposure, target engagement, and ecological safety are required before translation.

## 9 Integrated model: pro-healing and pro-leak mucosal immune states

The pro-healing and pro-leak states are provisional, spatially resolved wound endotypes rather than fixed patient categories. They are defined by co-occurring local functions and tissue responses, not by a single taxon, cytokine, or circulating biomarker.

### 9.1 Healing is a temporal decision process

**Figure 2** organizes anastomotic healing as an overlapping sequence. Early neutrophil recruitment supports bacterial control and initial epithelial repair. Continued microbial exposure or failed resolution can prolong neutrophil oxidative and proteolytic activity. Macrophage functions likewise change from microbial clearance and inflammatory coordination toward efferocytosis, stromal support, angiogenesis, and matrix remodeling; a binary M1/M2 label cannot represent this progression. During proliferative repair, epithelial restitution, IL-22- and YAP-responsive programs, fibroblast activity, and vascular support gain importance. The remodeling decision then depends on whether collagen synthesis, organization, and preservation exceed microbial and host proteolysis.

**Figure 2:**
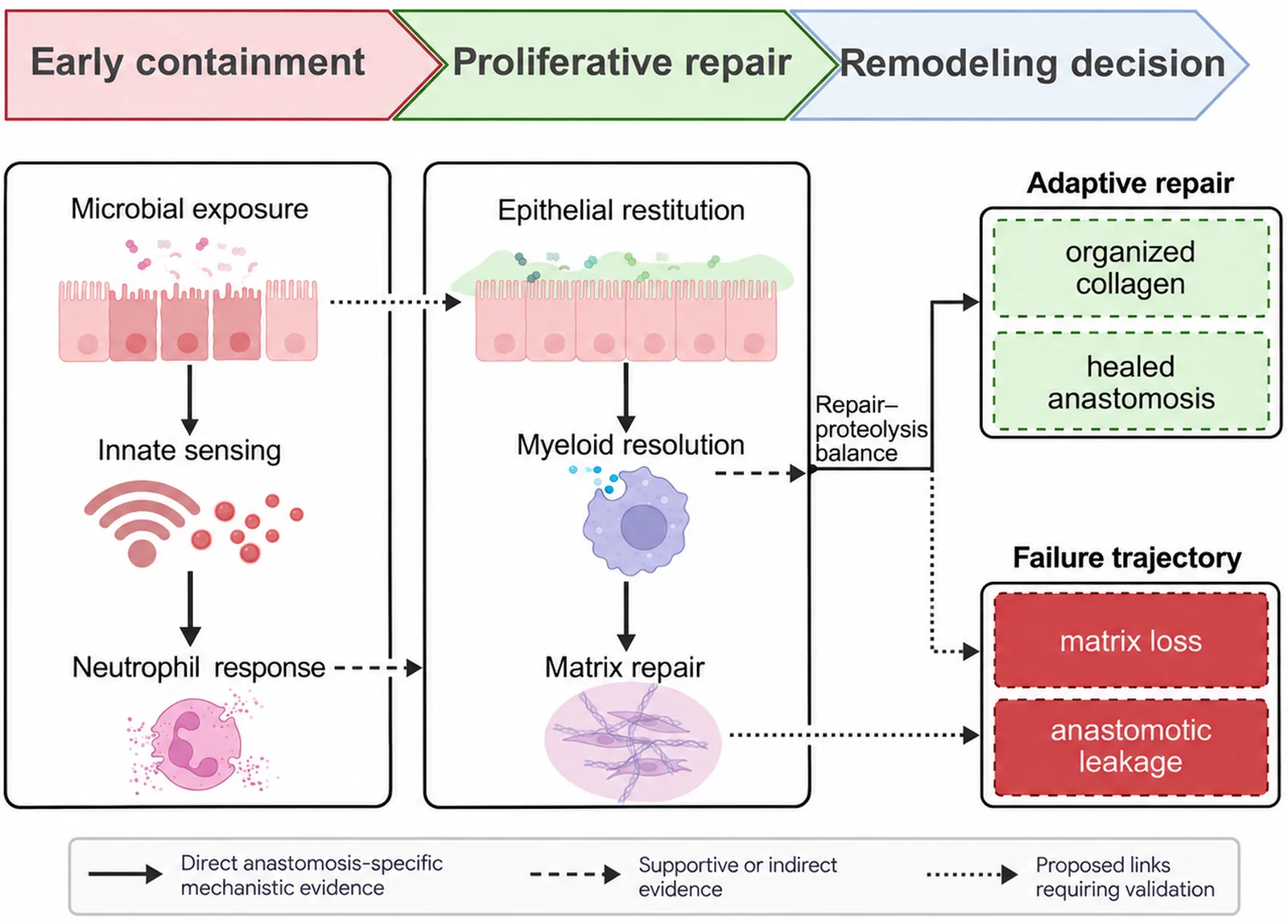
Temporal immune programs and remodeling decision in colorectal anastomotic healing. The phases overlap rather than forming discrete stages. Solid arrows indicate direct anastomosis-specific mechanistic evidence, dashed arrows indicate supportive or indirect evidence, and dotted arrows indicate proposed links requiring validation. The proposed balance between repair and proteolysis must be tested with temporally and spatially matched epithelial, immune, matrix, mechanical, and clinical endpoints.

Both states may coexist within different regions of the same wound. A repair-supportive region may transition toward proteolytic failure when microbial exposure persists, perfusion deteriorates, or inflammatory resolution fails; conversely, timely containment and restoration of the epithelial barrier may restrict a focal injury program. Directionality and thresholds remain hypotheses until confirmed by serial human sampling.

A practical validation study would pair intraoperative margin or stapled-doughnut tissue with early postoperative wound-proximal samples, prespecify the intended sampling window, and integrate microbial function, spatial immune state, epithelial continuity, protease activity, collagen architecture, and clinical leakage. Mechanical testing is appropriate for experimental models, whereas human studies should use validated structural and clinical surrogates.

## 10 Discussion: future research framework and local translation

### 10.1 Spatially resolved human studies

Human studies should now move from taxonomic association to spatially resolved function. Preoperative stool can characterize susceptibility, but intraoperative mucosa or tissue from both anastomotic ends is better positioned to measure wound-proximal organisms and virulence. When clinically safe, early postoperative samples should precede overt deterioration. Analyses should integrate microbial culture or metagenomics with virulence-gene expression, enzyme activity, spatial immunophenotyping, collagen imaging, perfusion, and structural outcomes. Antibiotics, diet, bowel preparation, tumor location, neoadjuvant treatment, platform, batch, comorbidity, diversion, and leak timing should be prespecified. Relative-abundance analyses should respect compositional structure, and prediction models should report event counts, validation, calibration, and clinical utility (19, 21, 31).

Wound-immune endotypes should be defined by measurable programs rather than isolated taxa or plasma cytokines. Two provisional examples are especially testable: a proteolytic-neutrophil endotype, characterized by high local collagenase activity or GelE/SprE/plasminogen activation together with sustained neutrophil-MMP9 or elastase activity and collagen disorganization; and a repair-deficient epithelial endotype, characterized by low AhR-ligand exposure, weak IL-22 target engagement, persistent epithelial defects, and impaired collagen replacement. For inflammasome-related endotypes, evidence would need to include caspase-1 and gasdermin-D processing, mature cytokine localization, cell source, and a temporally ordered structural consequence.

### 10.2 Mechanism-matched interventions and local safety

For translation, candidate interventions are separated into three evidence tiers: perioperative measures already tested in human trials, strategies tested directly in experimental anastomosis models, and hypotheses supported mainly by other intestinal injury settings. Readiness for clinical evaluation refers to mechanism-embedded, regimen-specific investigation rather than routine clinical adoption. Candidate strategies and the evidence required before clinical adoption are summarized in **Table 4**. 

**Table 4:** Immunology-guided translational strategies and evidence required before leak-prevention use.

Strategy	Immune or microbial target	Current evidence	Requirement before clinical adoption
Standard bowel preparation and oral antibiotics	Broad microbial load and perioperative ecology	Clinical benefit for infection; heterogeneous leakage effect	Regimen-specific efficacy, stewardship, resistance, ecological recovery, and mechanistic substudy
Repair-supportive metabolites or prebiotics	AhR–IL-22, epithelial metabolism, community function	Pathway-specific and dietary experimental evidence	Defined compound or formulation, local exposure, target engagement, tumor-related and infectious safety
Defined strains or consortia	Competitive exclusion, metabolite production, immune modulation	Community transfer and selected strain-rescue studies	Product identity, expressed function, engraftment, translocation risk, and leak-powered randomized trial
Anti-virulence or microbial-enzyme inhibition	Collagenases, GelE/SprE, beta-glucuronidase, wound-induced virulence	Direct prevention in sensitized animal models	Human target prevalence, local delivery, resistance, toxicology, and target-engagement assay
Local antifibrinolytic approach	Pathogen-mediated plasminogen activation	Structural rescue in pathogen-driven models	Confirmation of active pathway in human tissue, thrombosis and healing safety, randomized evaluation
Host protease or neutrophil modulation	MMP9, elastase, sustained neutrophilic inflammation	Direct mechanistic evidence but stage-dependent physiological roles	Spatial overactivity, narrow therapeutic window, preserved defense, epithelial closure, and mechanical strength
Inflammasome-directed therapy	NLRP3, NLRP6, or NLRC4	Candidate rationale without decisive anastomosis mediation	Pathway-specific human activation, cell source, perturbation/rescue, and infection safety; not currently justified

Randomized evidence shows that antimicrobial effects vary by operation, anatomical site, and regimen. In MOBILE2, mechanical bowel preparation plus oral neomycin and metronidazole reduced complications, surgical-site infection, and anastomotic dehiscence after rectal resection compared with mechanical preparation plus placebo (101). In the SELECT trial, selective digestive decontamination reduced infectious complications but did not significantly reduce leakage and the trial stopped when superiority for the primary endpoint was no longer attainable (102). A small single-center trial of local antibiotic decontamination reported fewer rectal leaks but was stopped after an interim analysis at 80 of 280 planned participants (103). These results should not be pooled into a claim that any bowel preparation or local antibiotic regimen is universally protective.

Potential harms include antimicrobial resistance, C. difficile infection, delayed recovery of anaerobic colonization resistance, selection of organisms that complicate later infection, and invasive infection or translocation associated with live microbial products. Probiotics, synbiotics, prebiotics, rational consortia, and fecal microbiota transplantation therefore require defined product identity, functional readouts, engraftment or metabolite confirmation, surveillance for resistance and infection, and adequately powered leak endpoints before routine use.

Mechanism-matched interventions are most defensible when they target an expressed local function, such as microbial collagenase, beta-glucuronidase in an exposure-defined context, pathogen-mediated plasminogen activation, sustained neutrophil proteolysis, or a defined repair-supportive metabolite (12, 61, 62, 104). Local delivery could be achieved through anti-virulence approaches or immunomodulatory biomaterials, including ILC3/IL-22-associated patches and VEGF-releasing staples, but composite materials must still show which mediator is necessary for the structural benefit (49, 89). Host-directed inhibition requires particular caution because MMPs, neutrophils, innate sensors, cytokines, and fibrinolytic pathways participate in antimicrobial defense and normal remodeling. A credible intervention should therefore demonstrate local target engagement, preserved microbial containment, restored epithelial or matrix architecture, improved mechanical competence, and ecological safety.

Interventions supported mainly by colitis, infection, organoid, or superficial-wound models—including several inflammasome, efferocytosis, lymphoid, fungal, and metabolite pathways—first require anastomosis-specific evidence that the proposed target is active in the relevant tissue compartment and postoperative window. A narrower claim or observational human target-engagement study is more appropriate than an efficacy trial when this prerequisite is absent.

### 10.3 Minimum causal evidence chain and review limitations

A minimum causal evidence chain should proceed in linked steps. First, detect a wound-relevant function before leakage and confirm that it is expressed locally. Second, reproduce the phenotype using a transferred community, defined organism, enzyme, or metabolite. Third, identify the immune mediator in the same spatial unit and test loss of function and rescue. Finally, evaluate a mechanism-matched intervention with target-engagement, structural, clinical, and ecological-safety endpoints. Prevention, intraoperative local therapy, and postoperative rescue should be studied as distinct windows because the same mediator can support early containment yet amplify later injury. Provisional operational definitions and validation requirements for spatial wound-immune states are summarized in **Table 5**.

**Table 5:** Provisional operational definitions and validation requirements for spatial wound-immune states.

Provisional state	Tissue compartment	Candidate measurements	Sampling window	Interpretation boundary
Pro-healing	Epithelial edge, lamina propria, submucosa	Epithelial continuity; local AhR target engagement; IL-22-associated epithelial response; efferocytosis or resolution markers; organized collagen	Intraoperative baseline; postoperative days 1-3; proliferative or remodeling window	Barrier improvement does not establish mechanical recovery; measurements require spatial colocalization and prospective outcome validation
Pro-leak	Unresolved wound edge, submucosal matrix, peri-anastomotic fluid	Expressed microbial virulence; collagenase or plasmin activity; persistent neutrophils; MMP9 or elastase activity; collagen disorganization	Before overt leakage whenever feasible; serial early postoperative sampling	Detection after leakage may reflect contamination or reverse causation; no single measurement defines the state

This review is limited by its mechanistic narrative design. SANRA informed reporting, but the search and selection process was not a systematic review and PRISMA was therefore not used as a claim of systematic completeness (17, 18). Human microbiome reports were interpreted using STORMS domains, animal reports with attention to ARRIVE 2.0, and prediction studies with PROBAST principles (19–21). Direct human data linking anastomotic spatial immune states to later leakage remain scarce, and many macrophage, inflammasome, epithelial-repair, fungal, and metabolite mechanisms derive from animal models, organoids, colitis, infection, or biopsy wounds.

These limitations define the scope and falsifiability of the proposed model. The framework separates human association, direct microbial-function measurement, anastomosis-specific experimental causation, and human intervention evidence; it does not assign equal weight to each category.

## 11 Conclusion

Colorectal anastomotic healing is a spatial mucosal immune process in which luminal microbial function, the epithelial wound edge, recruited immune cells, the submucosal matrix, and vascular support must remain coordinated. The most compelling direct evidence concerns microbial and host proteolytic functions that reduce collagen integrity; the complementary repair arm is biologically plausible and partly pathway-resolved but remains less direct and more heterogeneous.

The immediate priority is a spatially resolved human program that pairs local microbial function with immune-cell state, epithelial continuity, protease activity, collagen architecture, and clinically meaningful outcomes. Mechanism-matched trials should follow only after local target engagement and ecological safety have been established.

## References

[1] Rahbari NN Weitz J Hohenberger W Heald RJ Moran B Ulrich A . Definition and grading of anastomotic leakage following anterior resection of the rectum: A proposal by the International Study Group of Rectal Cancer. Surgery. (2010) 147:339–51. doi: 10.1016/j.surg.2009.10.012 20004450

[2] Helliwell JA Quyn A Jayne DG . Anastomotic leak after colorectal surgery: current landscape and future directions. Br J Surg. (2026) 113:znag041. doi: 10.1093/bjs/znag041 41902520

[3] Van Helsdingen CPM Wildeboer ACL Zafeiropoulou K Jongen ACHM Bosmans JWAM Gallé C . Histology and transcriptome insights into the early processes of intestinal anastomotic healing: a rat model. BJS Open. (2023) 7:zrad099. doi: 10.1093/bjsopen/zrad099 37855751 PMC10586197

[4] Weber M-C Clees Z Buck A Fischer A Steffani M Wilhelm D . Role of the serosa in intestinal anastomotic healing: insights from in-depth histological analysis of human and murine anastomoses. BJS Open. (2024) 8:zrae108. doi: 10.1093/bjsopen/zrae108 39230923 PMC11373408

[5] Sengul H Bantavi V Gloeck L Li Yim AYF Leven P Efferz P . Layer-specific molecular signatures of colon anastomotic healing and leakage in mice. Mol Med. (2025) 31:124. doi: 10.1186/s10020-025-01167-9 40169980 PMC11959837

[6] Shogan BD Belogortseva N Luong PM Zaborin A Lax S Bethel C . Collagen degradation and MMP9 activation by *Enterococcus faecalis* contribute to intestinal anastomotic leak. Sci Transl Med. (2015) 7(286):286ra68. doi: 10.1126/scitranslmed.3010658 25947163 PMC5027898

[7] Shi S Liu Y Wang Z Jin X Yan W Guo X . Fusobacterium nucleatum induces colon anastomosis leak by activating epithelial cells to express MMP9. Front Microbiol. (2022) 13:1031882. doi: 10.3389/fmicb.2022.1031882 36590433 PMC9794562

[8] Wei Z Bao L Li B Yang J Wang J Xu F . Fusobacterium nucleatum promotes anastomotic leakage by activating epithelial cells through the NOD1/RIPK2/ERK signalling pathway to drive IL‐1β‐induced neutrophil chemotaxis and collagen degradation. Clin Trans Med. (2025) 15:e70262. doi: 10.1002/ctm2.70262 40045503 PMC12128141

[9] Hyoju SK Adriaansens C Wienholts K Sharma A Keskey R Arnold W . Low-fat/high-fibre diet prehabilitation improves anastomotic healing via the microbiome: an experimental model. J Br Surg. (2020) 107:743–55. doi: 10.1002/bjs.11388 31879948 PMC7875206

[10] Hajjar R Oliero M Cuisiniere T Fragoso G Calvé A Djediai S . Improvement of colonic healing and surgical recovery with perioperative supplementation of inulin and galacto-oligosaccharides. Clin Nutr. (2021) 40:3842–51. doi: 10.1016/j.clnu.2021.04.032 34130031

[11] Hajjar R Gonzalez E Fragoso G Oliero M Alaoui AA Calvé A . Gut microbiota influence anastomotic healing in colorectal cancer surgery through modulation of mucosal proinflammatory cytokines. Gut. (2023) 72:1143–54. doi: 10.1136/gutjnl-2022-328389 36585238

[12] Bantavi V Gloeck L Leven P Zafeiropoulou K Welting O Sengül H . Tryptophan indole metabolites reduce anastomotic leakage through aryl hydrocarbon receptor-driven interleukin-22 production. Cell Mol Gastroenterol Hepatol. (2026) 20:101756. doi: 10.1016/j.jcmgh.2026.101756 41707749 PMC13094658

[13] Cashin PH Artursson S Sköldberg F Melhus Å . Association of the gut microbiome to colorectal anastomotic leakage: systematic review. BJS Open. (2026) 10:zrag005. doi: 10.1093/bjsopen/zrag005 41957947 PMC13065240

[14] Lianos GD Frountzas M Kyrochristou ID Sakarellos P Tatsis V Kyrochristou GD . What is the role of the gut microbiota in anastomotic leakage after colorectal resection? A scoping review of clinical and experimental studies. JCM. (2024) 13:6634. doi: 10.3390/jcm13226634 39597778 PMC11594793

[15] Helliwell JA Sciberras P Dosis A Burke J Chilton CH Wood HM . Modulation of the gut microbiota as a novel strategy to prevent anastomotic leak after colorectal surgery: systematic scoping review. Colorectal Dis. (2026) 28:e70472. doi: 10.1111/codi.70472 42083117 PMC13139701

[16] Sun S Long F Su B Chen J Luo Y Zhong Y . The gut microbiome in colorectal anastomotic leakage: from mechanisms to precision. Front Med. (2026) 13:1781458. doi: 10.3389/fmed.2026.1781458 41877779 PMC13007679

[17] Baethge C Goldbeck-Wood S Mertens S . SANRA—a scale for the quality assessment of narrative review articles. Res Integrity Peer Rev. (2019) 4:5. doi: 10.1186/s41073-019-0064-8 30962953 PMC6434870

[18] Page MJ McKenzie JE Bossuyt PM Boutron I Hoffmann TC Mulrow CD . The PRISMA 2020 statement: An updated guideline for reporting systematic reviews. BMJ. (2021) 372:n71. doi: 10.1136/bmj.n71 33782057 PMC8005924

[19] Mirzayi C Renson A Consortium GS Analysis M Society QC Zohra F . Reporting guidelines for human microbiome research: The STORMS checklist. Nat Med. (2021) 27:1885–92. doi: 10.1038/s41591-021-01552-x 34789871 PMC9105086

[20] Percie du Sert N Hurst V Ahluwalia A Alam S Avey MT Baker M . The ARRIVE guidelines 2.0: Updated guidelines for reporting animal research. PloS Biol. (2020) 18:e3000410. doi: 10.1371/journal.pbio.3000410 32663219 PMC7360023

[21] Wolff RF Moons KGM Riley RD Whiting PF Westwood M Collins GS . PROBAST: A tool to assess the risk of bias and applicability of prediction model studies. Ann Intern Med. (2019) 170:51–8. doi: 10.7326/M18-1376 30596875

[22] Johansson MEV Phillipson M Petersson J Velcich A Holm L Hansson GC . The inner of the two Muc2 mucin-dependent mucus layers in colon is devoid of bacteria. Proc Natl Acad Sci. (2008) 105:15064–9. doi: 10.1073/pnas.0803124105 18806221 PMC2567493

[23] Birchenough GMH Nyström EEL Johansson MEV Hansson GC . A sentinel goblet cell guards the colonic crypt by triggering Nlrp6-dependent Muc2 secretion. Science. (2016) 352:1535–42. doi: 10.1126/science.aaf7419 27339979 PMC5148821

[24] Shogan BD Smith DP Christley S Gilbert JA Zaborina O Alverdy JC . Intestinal anastomotic injury alters spatially defined microbiome composition and function. Microbiome. (2014) 2:35. doi: 10.1186/2049-2618-2-35 25250176 PMC4171717

[25] Komen N Slieker J Willemsen P Mannaerts G Pattyn P Karsten T . Polymerase chain reaction for enterococcus faecalis in drain fluid: The first screening test for symptomatic colorectal anastomotic leakage. The appeal-study: analysis of parameters predictive for evident anastomotic leakage. Int J Colorectal Dis. (2013) 29:15–21. doi: 10.1007/s00384-013-1776-8 24122105

[26] Helliwell JA Chilton CH Young C Clark EV Wilkinson L Fuentes Balaguer A . Perioperative changes in the microbiome during rectal cancer surgery: exploratory analysis of the National Institute for Health and Care Research (NIHR) IntAct trial. Br J Surg. (2025) 112:znaf199. doi: 10.1093/bjs/znaf199 41025606 PMC12481238

[27] Hoedt EC Carroll GM Stephensen BD Morrison M Vishnoi V Cuskelly A . Preoperative antibiotics and mechanical bowel preparation impact the colonic mucosa–associated microbiota but not anastomotic leak rate after colorectal resection. Dis Colon Rectum. (2025) 68:875–86. doi: 10.1097/DCR.0000000000003775 40214103

[28] Wienholts K Petersen AE Van Helsdingen CPM Talboom K Davids M De Wilt JHW . Perioperative faecal microbiome dynamics in patients undergoing rectal cancer surgery in the IMARI-trial. BJS Open. (2026) 10:zrag046. doi: 10.1093/bjsopen/zrag046 42213482 PMC13220757

[29] Boatman S Khan MH Ganesan N Nalluri-Butz H Kohn J Troester A . Anastomotic leak occurs independently from microbiota shifts associated with surgical bowel preparation. Sci Rep. (2024) 14:21711. doi: 10.1038/s41598-024-72520-x 39289419 PMC11408509

[30] De Simoni O Scarpa M Cavallin F Kotsafti A Marchegiani F Stepanyan A . IMMUNOREACT 4: Peritumoral microenvironment associated with anastomotic leaks after surgery for rectal cancer. Cancers. (2026) 18:571. doi: 10.3390/cancers18040571 41749825 PMC12938832

[31] Gloor GB Macklaim JM Pawlowsky-Glahn V Egozcue JJ . Microbiome datasets are compositional: and this is not optional. Front Microbiol. (2017) 8. doi: 10.3389/fmicb.2017.02224 29187837 PMC5695134

[32] Savage FJ Lacombe DLP Boulos PB Hembry RM . Role of matrix metalloproteinases in healing of colonic anastomosis. Dis Colon Rectum. (1997) 40:962–70. doi: 10.1007/bf02051206 9269815

[33] Strowitzki MJ Kimmer G Wehrmann J Ritter AS Radhakrishnan P Opitz VM . Inhibition of HIF-prolyl hydroxylases improves healing of intestinal anastomoses. JCI Insight. (2021) 6(8):e139191. doi: 10.1172/jci.insight.139191 33784253 PMC8119215

[34] Huisman DE Reudink M van Rooijen SJ Bootsma BT van de Brug T Stens J . LekCheck: A prospective study to identify perioperative modifiable risk factors for anastomotic leakage in colorectal surgery. Ann Surg. (2020) 275:e189–97. doi: 10.1097/sla.0000000000003853 32511133 PMC8683256

[35] Faber RA Meijer RPJ Droogh DHM Jongbloed JJ Bijlstra OD Boersma F . Indocyanine green near-infrared fluorescence bowel perfusion assessment to prevent anastomotic leakage in minimally invasive colorectal surgery (AVOID): a multicentre, randomised, controlled, phase 3 trial. Lancet Gastroenterol Hepatol. (2024) 9:924–34. doi: 10.1016/S2468-1253(24)00198-5 39151436

[36] Jayne D Croft J Corrigan N Quirke P Cahill RA Ainsworth G . Intraoperative fluorescence angiography with indocyanine green to prevent anastomotic leak in rectal cancer surgery (IntAct): an unblinded randomised controlled trial. Lancet Gastroenterol Hepatol. (2025) 10:806–17. doi: 10.1016/S2468-1253(25)00101-3 40690925

[37] Jacobson RA Wienholts K Williamson AJ Gaines S Hyoju S Van Goor H . *Enterococcus faecalis* exploits the human fibrinolytic system to drive excess collagenolysis: implications in gut healing and identification of druggable targets. Am J Physiology-Gastrointestinal Liver Physiol. (2020) 318:G1–9. doi: 10.1152/ajpgi.00236.2019 31604031 PMC6985841

[38] Winter M Heitplatz B Koppers N Mohr A Bungert AD Juratli MA . The impact of phase-specific macrophage depletion on intestinal anastomotic healing. Cells. (2023) 12:1039. doi: 10.3390/cells12071039 37048112 PMC10093464

[39] Jin X Liu Y Yan W Shi S Liu L Lin B . Gut microbiota from nCAL patients promotes colon anastomotic healing by inducing collagen synthesis in epithelial cells. J Gastro Hepatol. (2022) 37:1756–67. doi: 10.1111/jgh.15946 35849493

[40] Pickert G Neufert C Leppkes M Zheng Y Wittkopf N Warntjen M . STAT3 links IL-22 signaling in intestinal epithelial cells to mucosal wound healing. J Exp Med. (2009) 206:1465–72. doi: 10.1084/jem.20082683 19564350 PMC2715097

[41] Miyoshi H VanDussen KL Malvin NP Ryu SH Wang Y Sonnek NM . Prostaglandin E2 promotes intestinal repair through an adaptive cellular response of the epithelium. EMBO J. (2016) 36:5–24. doi: 10.15252/embj.201694660 27797821 PMC5210160

[42] Yui S Azzolin L Maimets M Pedersen MT Fordham RP Hansen SL . YAP/TAZ-dependent reprogramming of colonic epithelium links ECM remodeling to tissue regeneration. Cell Stem Cell. (2018) 22:35–49.e7. doi: 10.1016/j.stem.2017.11.001 29249464 PMC5766831

[43] Rakoff-Nahoum S Paglino J Eslami-Varzaneh F Edberg S Medzhitov R . Recognition of commensal microflora by toll-like receptors is required for intestinal homeostasis. Cell. (2004) 118:229–41. doi: 10.1016/j.cell.2004.07.002 15260992

[44] Alam A Leoni G Quiros M Wu H Desai C Nishio H . The microenvironment of injured murine gut elicits a local pro-restitutive microbiota. Nat Microbiol. (2016) 1:15021. doi: 10.1038/nmicrobiol.2015.21 27571978 PMC5076466

[45] Okada M Bothin C Kanazawa K Midtvedt T . Experimental study of the influence of intestinal flora on the healing of intestinal anastomoses. J Br Surg. (1999) 86:961–5. doi: 10.1046/j.1365-2168.1999.01161.x 10417574

[46] Bachmann R Van Hul M Baldin P Léonard D Delzenne NM Belzer C . Akkermansia muciniphila reduces peritonitis and improves intestinal tissue wound healing after a colonic transmural defect by a MyD88-dependent mechanism. Cells. (2022) 11:2666. doi: 10.3390/cells11172666 36078075 PMC9454966

[47] Lindemans CA Calafiore M Mertelsmann AM O’Connor MH Dudakov JA Jenq RR . Interleukin-22 promotes intestinal-stem-cell-mediated epithelial regeneration. Nature. (2015) 528:560–4. doi: 10.1038/nature16460 26649819 PMC4720437

[48] Zha J-M Li H-S Lin Q Kuo W-T Jiang Z-H Tsai P-Y . Interleukin 22 expands transit-amplifying cells while depleting Lgr5+ stem cells via inhibition of Wnt and Notch signaling. Cell Mol Gastroenterol Hepatol. (2019) 7:255–74. doi: 10.1016/j.jcmgh.2018.09.006 30686779 PMC6352747

[49] Zhong H-J Zhou Y Zhao J-W Chen W-R Zhan N-Y Zhan Y-Q . Enhancement of gastrointestinal anastomosis healing via a small intestinal submucosa bio-patch: modulating IL-22 secretion by type 3 innate lymphoid cells and microbial structures. Front Bioeng Biotechnol. (2026) 14:1752619. doi: 10.3389/fbioe.2026.1752619 41768136 PMC12946001

[50] Stumpf M Klinge U Wilms A Zabrocki R Rosch R Junge K . Changes of the extracellular matrix as a risk factor for anastomotic leakage after large bowel surgery. Surgery. (2005) 137:229–34. doi: 10.1016/j.surg.2004.07.011 15674206

[51] Ågren MS Andersen TL Mirastschijski U Syk I Schiødt CB Surve V . Action of matrix metalloproteinases at restricted sites in colon anastomosis repair: An immunohistochemical and biochemical study. Surgery. (2006) 140:72–82. doi: 10.1016/j.surg.2005.12.013 16857445

[52] Türlü C Willumsen N Marando D Schjerling P Biskup E Hannibal J . A human cellular model for colorectal anastomotic repair: The effect of localization and transforming growth factor-β1 treatment on collagen deposition and biomarkers. Int J Mol Sci. (2021) 22:1616. doi: 10.3390/ijms22041616 33562728 PMC7914853

[53] Krarup P-M Eld M Jorgensen LN Hansen MB Ågren MS . Selective matrix metalloproteinase inhibition increases breaking strength and reduces anastomotic leakage in experimentally obstructed colon. Int J Colorectal Dis. (2017) 32:1277–84. doi: 10.1007/s00384-017-2857-x 28717842

[54] Edomskis P Goudberg MR Sparreboom CL Menon AG Wolthuis AM D’Hoore A . Matrix metalloproteinase-9 in relation to patients with complications after colorectal surgery: a systematic review. Int J Colorectal Dis. (2021) 36:1–10. doi: 10.1007/s00384-020-03724-6 32865714 PMC7782374

[55] Jørgensen AB Almer L Samaniego Castruita JA Pedersen MS Kirkby NS Jensen EA . The baseline fecal microbiome differs in patients with and without anastomotic leakage after colorectal cancer surgery. Heliyon. (2024) 10:e40616. doi: 10.1016/j.heliyon.2024.e40616 39687136 PMC11647948

[56] Jørgensen AB Jonsson I Friis-Hansen L Brandstrup B . Collagenase-producing bacteria are common in anastomotic leakage after colorectal surgery: a systematic review. Int J Colorectal Dis. (2023) 38:275. doi: 10.1007/s00384-023-04562-y 38038731 PMC10692267

[57] Anderson DI Keskey R Ackerman MT Zaborina O Hyman N Alverdy JC . *Enterococcus faecalis* is associated with anastomotic leak in patients undergoing colorectal surgery. Surg Infections. (2021) 22:1047–51. doi: 10.1089/sur.2021.147 34255574 PMC8851212

[58] Olivas AD Shogan BD Valuckaite V Zaborin A Belogortseva N Musch M . Intestinal tissues induce an SNP mutation in Pseudomonas aeruginosa that enhances its virulence: Possible role in anastomotic leak. PloS One. (2012) 7:e44326. doi: 10.1371/journal.pone.0044326 22952955 PMC3432121

[59] Christley S Shogan B Levine Z Koo H Guyton K Owens S . Comparative genetics of Enterococcus faecalis intestinal tissue isolates before and after surgery in a rat model of colon anastomosis. PloS One. (2020) 15:e0232165. doi: 10.1371/journal.pone.0232165 32343730 PMC7188289

[60] Hernández‐González PI Barquín J Ortega‐Ferrete A Patón V Ponce‐Alonso M Romero‐Hernández B . Anastomotic leak in colorectal cancer surgery: Contribution of gut microbiota and prediction approaches. Colorectal Dis. (2023) 25:2187–97. doi: 10.1111/codi.16733 37743721

[61] Jacobson RA Williamson AJ Wienholts K Gaines S Hyoju S Van Goor H . Prevention of anastomotic leak via local application of tranexamic acid to target bacterial-mediated plasminogen activation: A practical solution to a complex problem. Ann Surg. (2021) 274:e1038–46. doi: 10.1097/SLA.0000000000003733 31851007 PMC7875208

[62] Hyoju SK Klabbers RE Aaron M Krezalek MA Zaborin A Wiegerinck M . Oral polyphosphate suppresses bacterial collagenase production and prevents anastomotic leak due to Serratia marcescens and Pseudomonas aeruginosa. Ann Surg. (2018) 267:1112–8. doi: 10.1097/SLA.0000000000002167 28166091 PMC5540820

[63] Wiegerinck M Hyoju SK Mao J Zaborin A Adriaansens C Salzman E . Novel *de novo* synthesized phosphate carrier compound ABA-PEG20k-Pi20 suppresses collagenase production in *Enterococcus faecalis* and prevents colonic anastomotic leak in an experimental model. Br J Surg. (2018) 105:1368–76. doi: 10.1002/bjs.10859 29658991 PMC6827486

[64] Sumagin R Brazil JC Nava P Nishio H Alam A Luissint AC . Neutrophil interactions with epithelial-expressed ICAM-1 enhances intestinal mucosal wound healing. Mucosal Immunol. (2016) 9:1151–62. doi: 10.1038/mi.2015.135 26732677 PMC4935657

[65] Weber DA Sumagin R McCall IC Leoni G Neumann PA Andargachew R . Neutrophil-derived JAML inhibits repair of intestinal epithelial injury during acute inflammation. Mucosal Immunol. (2014) 7:1221–32. doi: 10.1038/mi.2014.12 24621992 PMC4340686

[66] Miranda J Fink DJ Wilson ZS Hilgarth R Nusrat A Parkos CA . The protein tyrosine phosphatase CD45 promotes PMN transepithelial migration, antimicrobial function and colonic mucosal repair. JCI Insight. (2026) 11(15):e203438. doi: 10.1172/jci.insight.203438 42334869 PMC13463622

[67] Witte MB Saupe J Reiner J Bannert K Schafmayer C Lamprecht G . Ileocolonic healing after small ileocecal resection in mice: NOD2 deficiency impairs anastomotic healing by local mechanisms. JCM. (2023) 12:3601. doi: 10.3390/jcm12103601 37240707 PMC10219437

[68] Rauch I Deets KA Ji DX Von Moltke J Tenthorey JL Lee AY . NAIP-NLRC4 inflammasomes coordinate intestinal epithelial cell expulsion with eicosanoid and IL-18 release via activation of caspase-1 and -8. Immunity. (2017) 46:649–59. doi: 10.1016/j.immuni.2017.03.016 28410991 PMC5476318

[69] Buitrago-Ruiz M Arias-Sánchez C Asensio-López MM Martínez-García JJ Soria-Aledo V Valero-Navarro G . Gut microbiota and postoperative complications in colorectal surgery and its potential association with intestinal permeability and NLRP6 inflammasome. Front Immunol. (2026) 16:1701650. doi: 10.3389/fimmu.2025.1701650 41668765 PMC12884971

[70] Elinav E Strowig T Kau AL Henao-Mejia J Thaiss CA Booth CJ . NLRP6 inflammasome regulates colonic microbial ecology and risk for colitis. Cell. (2011) 145:745–57. doi: 10.1016/j.cell.2011.04.022 21565393 PMC3140910

[71] Mamantopoulos M Ronchi F Van Hauwermeiren F Vieira-Silva S Yilmaz B Martens L . Nlrp6- and ASC-dependent inflammasomes do not shape the commensal gut microbiota composition. Immunity. (2017) 47:339–348.e4. doi: 10.1016/j.immuni.2017.07.011 28801232

[72] Normand S Delanoye-Crespin A Bressenot A Huot L Grandjean T Peyrin-Biroulet L . Nod-like receptor pyrin domain-containing protein 6 (NLRP6) controls epithelial self-renewal and colorectal carcinogenesis upon injury. Proc Natl Acad Sci USA. (2011) 108:9601–6. doi: 10.1073/pnas.1100981108 21593405 PMC3111299

[73] Samperio Ventayol P Geiser P Di Martino ML Florbrant A Fattinger SA Walder N . Bacterial detection by NAIP/NLRC4 elicits prompt contractions of intestinal epithelial cell layers. Proc Natl Acad Sci USA. (2021) 118:e2013963118. doi: 10.1073/pnas.2013963118 33846244 PMC8072224

[74] Wlodarska M Thaiss CA Nowarski R Henao-Mejia J Zhang J-P Brown EM . NLRP6 inflammasome orchestrates the colonic host-microbial interface by regulating goblet cell mucus secretion. Cell. (2014) 156:1045–59. doi: 10.1016/j.cell.2014.01.026 24581500 PMC4017640

[75] Zaki M Boyd KL Vogel P Kastan MB Lamkanfi M Kanneganti T-D . The NLRP3 inflammasome protects against loss of epithelial integrity and mortality during experimental colitis. Immunity. (2010) 32:379–91. doi: 10.1016/j.immuni.2010.03.003 20303296 PMC2982187

[76] Seno H Miyoshi H Brown SL Geske MJ Colonna M Stappenbeck TS . Efficient colonic mucosal wound repair requires Trem2 signaling. Proc Natl Acad Sci USA. (2009) 106:256–61. doi: 10.1073/pnas.0803343106 19109436 PMC2629230

[77] Quiros M Nishio H Neumann PA Siuda D Brazil JC Azcutia V . Macrophage-derived IL-10 mediates mucosal repair by epithelial WISP-1 signaling. J Clin Invest. (2017) 127:3510–20. doi: 10.1172/JCI90229 28783045 PMC5669557

[78] Domanska D Majid U Karlsen VT Merok MA Beitnes A-C Yaqub S . Single-cell transcriptomic analysis of human colonic macrophages reveals niche-specific subsets. J Exp Med. (2022) 219:e20211846. doi: 10.1084/jem.20211846 35139155 PMC8932544

[79] Binnebösel M Schuler T Klink CD Busch D Schöb DS Von Trotha KT . Influence of CD68+ macrophages and neutrophils on anastomotic healing following laparoscopic sigmoid resection due to diverticulitis. Int J Colorectal Dis. (2014) 29:681–8. doi: 10.1007/s00384-014-1855-5 24728515

[80] Xue J Schmidt SV Sander J Draffehn A Krebs W Quester I . Transcriptome-based network analysis reveals a spectrum model of human macrophage activation. Immunity. (2014) 40:274–88. doi: 10.1016/j.immuni.2014.01.006 24530056 PMC3991396

[81] Liu R Shen Y Wei Y Zhu C Yao X Gu X . The central role of macrophages in skeletal muscle regeneration: Phenotypic polarization, metabolic reprogramming, and therapeutic prospects. Biochem Pharmacol. (2026) 250:118025. doi: 10.1016/j.bcp.2026.118025 42069229

[82] Berlin P Fischer A Bergmann-Ewert W Kordt M Schafmayer C Lamprecht G . Perioperative anti-α4β7 integrin blockade alters wound cell infiltrate but not the functional outcome of ileocecal anastomoses in mice with Crohn’s-like ileitis. Inflammatory Bowel Dis. (2025) 31:3385–97. doi: 10.1093/ibd/izaf223 41157912 PMC12688080

[83] Bujko A Atlasy N Landsverk OJB Richter L Yaqub S Horneland R . Transcriptional and functional profiling defines human small intestinal macrophage subsets. J Exp Med. (2018) 215:441–58. doi: 10.1084/jem.20170057 29273642 PMC5789404

[84] Wright PB McDonald E Bravo-Blas A Baer HM Heawood A Bain CC . The mannose receptor (CD206) identifies a population of colonic macrophages in health and inflammatory bowel disease. Sci Rep. (2021) 11:19616. doi: 10.1038/s41598-021-98611-7 34608184 PMC8490356

[85] Hickey JW Becker WR Nevins SA Horning A Perez AE Zhu C . Organization of the human intestine at single-cell resolution. Nature. (2023) 619:572–84. doi: 10.1038/s41586-023-05915-x 37468586 PMC10356619

[86] Garrido-Trigo A Corraliza AM Veny M Dotti I Melón-Ardanaz E Rill A . Macrophage and neutrophil heterogeneity at single-cell spatial resolution in human inflammatory bowel disease. Nat Commun. (2023) 14:4506. doi: 10.1038/s41467-023-40156-6 37495570 PMC10372067

[87] Quiros M Feier D Birkl D Agarwal R Zhou DW García AJ . Resolvin E1 is a pro-repair molecule that promotes intestinal epithelial wound healing. Proc Natl Acad Sci USA. (2020) 117:9477–82. doi: 10.1073/pnas.1921335117 32300016 PMC7197018

[88] Meriwether D Jones AE Ashby JW Solorzano-Vargas RS Dorreh N Noori S . Macrophage COX2 mediates efferocytosis, resolution reprogramming, and intestinal epithelial repair. Cell Mol Gastroenterol Hepatol. (2022) 13:1095–120. doi: 10.1016/j.jcmgh.2022.01.002 35017061 PMC8873959

[89] Li R Sun Q Xu R Zhu R Lin J Tang Q . Bioactive-coated porous anastomotic staples enhance anastomotic healing. Bioact Mater. (2026) 60:40–54. doi: 10.1016/j.bioactmat.2026.01.005 41625499 PMC12856637

[90] Hendriks T Mastboom WJB . Healing of experimental intestinal anastomoses: Parameters for repair. Dis Colon Rectum. (1990) 33:891–901. doi: 10.1007/bf02051930 2209281

[91] Arron MNN Bluiminck S Ten Broek RPG Ederveen THA Alpert L Zaborina O . The combined effect of Western diet consumption and diclofenac administration alters the gut microbiota and promotes anastomotic leakage in the distal colon. Biomedicines. (2024) 12:2170. doi: 10.3390/biomedicines12102170 39457483 PMC11504258

[92] Guo MY Liu J Balmes P Yanta C Motamedi A Phang PT . Effects of diet and antibiotics on anastomotic healing: A mouse model study with varied dietary fiber and fat, and pre-operative antibiotics. Am J Surg. (2024) 235:115766. doi: 10.1016/j.amjsurg.2024.05.007 38777716

[93] Kelly CJ Zheng L Campbell EL Saeedi B Scholz CC Bayless AJ . Crosstalk between microbiota-derived short-chain fatty acids and intestinal epithelial HIF augments tissue barrier function. Cell Host Microbe. (2015) 17:662–71. doi: 10.1016/j.chom.2015.03.005 25865369 PMC4433427

[94] Kaiko GE Ryu SH Koues OI Collins PL Solnica-Krezel L Pearce EJ . The colonic crypt protects stem cells from microbiota-derived metabolites. Cell. (2016) 165:1708–20. doi: 10.1016/j.cell.2016.05.018 27264604 PMC5026192

[95] Longman RS Diehl GE Victorio DA Huh JR Galan C Miraldi ER . CX3CR1+ mononuclear phagocytes support colitis-associated innate lymphoid cell production of IL-22. J Exp Med. (2014) 211:1571–83. doi: 10.1084/jem.20140678 25024136 PMC4113938

[96] Romera-Hernández M Aparicio-Domingo P Papazian N Karrich JJ Cornelissen F Hoogenboezem RM . Yap1-driven intestinal repair is controlled by group 3 innate lymphoid cells. Cell Rep. (2020) 30:37–45.e3. doi: 10.1016/j.celrep.2019.11.115 31914395

[97] Monticelli LA Osborne LC Noti M Tran SV Zaiss DMW Artis D . IL-33 promotes an innate immune pathway of intestinal tissue protection dependent on amphiregulin–EGFR interactions. Proc Natl Acad Sci. (2015) 112:10762–7. doi: 10.1073/pnas.1509070112 26243875 PMC4553775

[98] Frede A Czarnewski P Monasterio G Tripathi KP Bejarano DA Ramirez Flores RO . B cell expansion hinders the stroma-epithelium regenerative cross talk during mucosal healing. Immunity. (2022) 55:2336–2351.e12. doi: 10.1016/j.immuni.2022.11.002 36462502

[99] Iliev ID Funari VA Taylor KD Nguyen Q Reyes CN Strom SP . Interactions between commensal fungi and the C-type lectin receptor dectin-1 influence colitis. Science. (2012) 336:1314–7. doi: 10.1126/science.1221789 22674328 PMC3432565

[100] Jain U Ver Heul AM Xiong S Gregory MH Demers EG Kern JT . *Debaryomyces* is enriched in Crohn’s disease intestinal tissue and impairs healing in mice. Science. (2021) 371:1154–9. doi: 10.1126/science.abd0919 33707263 PMC10114606

[101] Koskenvuo L Lunkka P Varpe P Hyöty M Satokari R Haapamäki C . Morbidity after mechanical bowel preparation and oral antibiotics prior to rectal resection. JAMA Surg. (2024) 159:606. doi: 10.1001/jamasurg.2024.0184 38506889 PMC10955353

[102] Abis GSA Stockmann HBAC Bonjer HJ van Veenendaal N van Doorn-Schepens MLM Budding AE . Randomized clinical trial of selective decontamination of the digestive tract in elective colorectal cancer surgery (SELECT trial). Br J Surg. (2019) 106:355–63. doi: 10.1002/bjs.11117 30802304

[103] Schardey HM Wirth U Strauss T Kasparek MS Schneider D Jauch KW . Prevention of anastomotic leak in rectal cancer surgery with local antibiotic decontamination: A prospective, randomized, double-blind, placebo-controlled single center trial. Int J Colorectal Dis. (2020) 35:847–57. doi: 10.1007/s00384-020-03544-8 32103326

[104] Yauw STK Arron M Lomme RMLM Van Den Broek P Greupink R Bhatt AP . Microbial glucuronidase inhibition reduces severity of diclofenac-induced anastomotic leak in rats. Surg Infections. (2018) 19:417–23. doi: 10.1089/sur.2017.245 29624485 PMC5961743

