# Supplementary material for: Microbial control of mucosal wound immunity in colorectal anastomotic healing: from barrier repair to matrix failure
Source: Front Immunol. 2026 Oct 21;17:1928237. doi: 10.3389/fimmu.2026.1928237 (PMC13732322; doi:10.3389/fimmu.2026.1928237)
Supplement: Supplementary file 1 [file Supplementaryfile1.docx]

**Supplementary Methods and Study Level Evidence Map**

*Microbial Control of Mucosal Wound Immunity in Colorectal Anastomotic Healing*

**Supplementary Methods**

**Search sources and dates.** PubMed/MEDLINE was searched through 28 June 2026. Europe PMC, OpenAlex, Crossref, and backward and forward citation searching were used to identify additional records and verify metadata.

**Representative broad search.** ("colorectal anastomosis" OR "intestinal anastomotic healing" OR "anastomotic leakage") AND (microbiome OR microbiota OR microbial virulence OR mucosal immunity OR epithelial restitution OR neutrophil OR macrophage OR MMP9 OR IL-22 OR AhR OR innate sensing OR inflammasome).

**Representative pathway searches.** Individual mediators or pathways were combined with anastomosis, intestinal wound healing, epithelial repair, collagen remodeling, bursting pressure, breaking strength, leakage, perturbation, inhibition, knockout, rescue, or target engagement. Examples included (AhR OR "aryl hydrocarbon receptor" OR IL-22) AND (anastomosis OR intestinal wound healing), and (MMP9 OR collagenase OR proteolysis OR plasminogen) AND (anastomotic healing OR anastomotic leakage).

**Selection of key studies.** Priority was given to studies that sampled anastomotic or peri-anastomotic tissue, directly measured microbial function, tested pathway perturbation or rescue, reported structural or mechanical endpoints, or provided prospective human or randomized intervention evidence. Evidence from colitis, infection, organoids, superficial wounds, or other organs was retained only when it addressed a specific mechanistic gap and was labeled indirect.

**Structured assessment.** STORMS informed review of sampling, perioperative metadata, specimen handling, sequencing and bioinformatic methods, batch effects, compositional analysis, and outcome reporting. ARRIVE 2.0 informed review of animal model, experimental unit, group size, allocation, blinding, exclusions, timing, and endpoints. PROBAST informed appraisal of participants, predictors, outcome definition, event counts, missing data, overfitting, validation, calibration, and clinical utility. These frameworks were not converted into numerical quality scores.

**Supplementary Table S1 Pivotal Study Level Evidence Map**

Sample sizes are reported for the principal in vivo or human comparison used in the review. Several mechanistic papers contained multiple experiments with different group sizes; these are indicated rather than collapsed into a misleading single total.

| **Ref** | **Study and model** | **Reported sample size** | **Sampling site and timing** | **Intervention or comparator** | **Principal outcome** | **Perturbation or rescue** | **Interpretation boundary** |
| --- | --- | --- | --- | --- | --- | --- | --- |
| 6 | Shogan et al. 2015; human resection samples and rat colon anastomosis | Human n=11; key rat comparisons n=5-20 per group | Proximal and distal resected human colon; rat anastomotic tissue after surgery | Collagenolytic E. faecalis strains; gelE/sprE mutant and complementation; topical antibiotic or MMP9 suppression | Collagen degradation, MMP9 activation, leak | Genetic perturbation and complementation; pharmacological rescue | Strong direct destructive-pathway evidence; human component is small and mainly functional/exploratory |
| 7 | Shi et al. 2022; CRC cohorts and rat colon anastomosis | Cohort 1: 10 AL and 10 non-AL; cohort 2: 16 AL and 29 non-AL; main rat comparison n=6/group | Human colorectal tissue; rat anastomosis, typically POD7 | F. nucleatum, heat-killed F. nucleatum, E. coli, or PBS; genistein rescue | Epithelial MMP9 induction, collagen I/III loss, leak | Pathway inhibition and rescue; no receptor-level genetic rescue | Supports an epithelial route to matrix injury; modest human cohorts and incomplete in vivo mediation |
| 8 | Wei et al. 2025; mouse colon anastomosis and epithelial systems | Core in vivo experiments generally n=6/group | Anastomotic tissue during early postoperative healing | F. nucleatum exposure with NOD1/RIPK2/ERK inhibition or knockdown and neutrophil perturbation | IL-1β-associated neutrophil recruitment, protease activity, collagen loss, impaired healing | Receptor/pathway and neutrophil perturbation | Direct pathway evidence, but small groups and incomplete resolution of IL-1β source |
| 12 | Bantavi et al. 2026; REVEAL human cohort and mouse anastomosis | Human cohort n=388; matched metabolomics 19 AL and 33 AH; mouse groups varied by assay, including IL-22 neutralization n=16/group and IL-22 rescue n=13 vs 10 | Preoperative human stool; mouse anastomotic tissue from hours to POD5 | AhR deficiency, epithelial AhR deletion, IL-22 neutralization, recombinant IL-22, diet and I3C | AhR target engagement, IL-22 production, anastomotic complication score and leakage | Genetic and pharmacological perturbation plus rescue | Most pathway-resolved repair-supportive evidence; human metabolite association is not a prevention trial |
| 38 | Winter et al. 2023; CD11b-DTR mouse distal-colon anastomosis | Histological/functional cohorts typically n=5-8/group; RNA-seq n=4/group | Anastomosis assessed during inflammatory, proliferative, and reparative phases | Timed CD11b-positive-cell depletion versus wild type | Inflammation, epithelial gap, angiogenesis, collagen, mechanical healing and phase-specific transcription | Temporal depletion; no rescue experiment | Demonstrates phase dependence but CD11b depletion is broader than a defined macrophage state |
| 49 | Zhong et al. 2026; mouse gastrointestinal anastomosis | n=7/group; n=6/group for most tissue analyses after one early death per group | Anastomotic tissue on POD5 | Small-intestinal-submucosa bio-patch versus sutures alone | Histology, ZO-1/occludin, IL-22-positive ILC3s, microbiota and metabolomics | Composite intervention; no pathway-specific rescue | Supports coordinated repair but does not isolate IL-22 or a microbial mediator |
| 61 | Jacobson et al. 2021; pathogen-driven mouse colorectal anastomosis | Core E. faecalis plus TXA experiment n=10/group | Anastomotic tissue after local postoperative treatment | Topical tranexamic acid versus vehicle in pathogen-exposed wounds | Plasminogen activation, collagenolysis, healing score and leak | Pharmacological rescue | Direct rescue in a sensitized pathogen-driven model; human target prevalence and safety remain unresolved |
| 62 | Hyoju et al. 2018; pathogen-exposed mouse colorectal anastomosis | 55 mice across principal protocol groups | Anastomotic tissue through POD10 | Oral PPi-6 versus water after S. marcescens or P. aeruginosa exposure | Bacterial collagenase and virulence traits, abscess and leak | Anti-virulence pharmacological rescue | Direct prevention in selected pathogen models; group-specific ecological effects require clinical validation |
| 63 | Wiegerinck et al. 2018; E. faecalis-exposed mouse colon anastomosis | 50 mice total; main groups n=10-15 | Anastomotic tissue on POD7 | ABA-PEG20k-Pi20, carrier control, or water | Collagenase suppression, healing score and leak | Anti-virulence rescue with carrier control | Direct preclinical rescue; formulation, exposure and safety remain to be established in humans |
| 67 | Witte et al. 2023; wild-type and Nod2-knockout limited ileocecal resection | 43 wild-type and 45 knockout mice randomized to vehicle or MDP; endpoint-specific groups varied | Ileocolonic anastomosis on POD5 | Nod2 genotype and muramyl dipeptide versus vehicle | Leak, bursting pressure, granulation tissue and matrix-turnover transcripts | Genetic deficiency; ligand treatment did not restore the main mechanical defect | Supports protective local NOD2 signaling; cell type and microbiome mediation remain unresolved |
